# Identifying type and determinants of missing items in quality of life questionnaires: Application to the SF-36 French version of the 2003 Decennial Health Survey

**DOI:** 10.1186/1477-7525-8-16

**Published:** 2010-02-03

**Authors:** Hugo Peyre, Joël Coste, Alain Leplège

**Affiliations:** 1 Biostatistics and Epidemiology Unit, Assistance Publique-Hôpitaux de Paris, Hôpital Cochin, Paris, France; 2 Nancy-Université, Université Paris-Descartes, Université Metz Paul Verlaine, Research unit APEMAC, EA 4360, Paris, France; 3 Department of History and Philosophy of Sciences, University of Paris Diderot - Paris 7, France

## Abstract

**Background:**

Missing items are common in quality of life (QoL) questionnaires and present a challenge for research in this field. The development of sound strategies of replacement and prevention requires accurate knowledge of their type and determinants.

**Methods:**

We used the 2003 French Decennial Health Survey of a representative sample of the general population -- including 22,620 adult subjects who completed the SF-36 questionnaire-- to test various socio-demographic, health status and QoL variables as potential predictors of missingness. We constructed logistic regression models for each SF-36 item to identify independent predictors and classify them according to Little and Rubin ("missing completely at random", "missing at random" and "missing not at random").

**Results:**

The type of missingness was missing at random for half of the items of the SF-36 and missing not at random for the others. None of the items were missing completely at random. Independent predictors of missingness were age, female sex, low scores on the SF-36 subscales and in some cases low educational level, occupation, nationality and poor health status.

**Conclusion:**

This study of the SF-36 shows that imputation of missing items is necessary and emphasizes several factors for missingness that should be considered in prevention strategies of missing data. Similar methodologies could be applied to item missingness in other QoL questionnaires.

## Background

In the field of quality of life (QoL) as in other research fields, missing data reduce the statistical power of studies and may cause selection biases if observations with missing values are excluded from the analysis [e.g. [1-3]]. However, the issue raised by incomplete data is of greater importance in QoL research because the items of questionnaires are usually aggregated to compute total (sub)scale score(s) and that any missing item of a subscale will cause the entire subscale score to be missing. Although there has been research addressing the replacement or "imputation" of missing items of QoL questionnaires, less attention has been paid to identifying their type (which nonetheless guides the choice of imputation methods [4-6]) and their determinants. It has repeatedly been shown that the best way of dealing with missing data is to minimize their amount i.e. *to prevent *them. A detailed understanding of their determinants is therefore required to devise appropriate prevention strategies. Some studies have suggested that determinants of missing data in QoL questionnaires are multiple and diverse, and may be socio-demographic (sex, age, educational level, marital status, etc.) or related to health status (some diseases or impairments, fatigue, etc.) [4,7-9]. The 2003 Decennial Health Survey of a large representative sample of the French population included 22,620 adult subjects who completed the SF-36 questionnaire; we used this survey to investigate a broad variety of socio-demographic, health status and QoL variables as potential predictors of item missingness in the SF-36 questionnaire.

## Methods

### Study population and data collection

The Decennial Health Survey was conducted by the French National Institute of Statistics and Economic Studies (INSEE), between October 2002 and October 2003; a representative sample of the French population was surveyed to provide data on the health status of this population and its demand for health services [10]. The sample included 25,482 subjects older than 18 years for whom standard socio-demographic and health status data were collected; some self-reported questionnaires including the CES-D [11] and the SF-36 [12,13] were also used. Of the subjects older than 18 years included, 2,862 did not complete the SF-36 ("missing forms": these subjects did not fill-in any question of the SF-36) such that our study addresses 22,620 subjects.

### The SF-36 questionnaire

The French SF-36 questionnaire [14,15] (version 1.3) used in the Decennial Health Survey was developed and validated as part of the International Quality of Life Assessment (IQOLA) project [16]. It is made up of 35 questions (Additional file 1) divided into eight scales: physical functioning (PF1 to PF10), role limitations relating to physical health (RP1 to RP4), bodily pain (BP1 and BP2), general health perceptions (GH1 to GH5), vitality (VT1 to VT4), social functioning (SF1 and SF2), role limitation relating to mental health (RE1 to RE3), and mental health (MH1 to MH5). One additional item assesses the health transition (HT). Each question is rated on an ordinal scale with between 2 to 6 categories. The score on each scale was calculated when more than the half of the items of the scale were available ("half item rule"); the score of the scale was the sum of the item scores further normalized to range from 0 to 100, with higher values representing better perceived QoL. The questionnaire is short and quick to administer (5-10 min) and well-adapted for studies in general populations.

### Strategy for identification of type and determinants of missingness

The type of missingness was defined according to Little and Rubin [17,18]: when the probability of missingness depends on what would have been the true answer, the item missingness is classified as being missing not at random (MNAR); when this probability does not depend on what would have been the true answer but depends on (observed) external covariates the item missingness is classified as being missing at random (MAR); when this probability is independent of (any observed) patient characteristics the item is classified as being missing completely at random (MCAR). The MNAR type is difficult to identify because the true value of the missing value is unknown [18]. In the case of missing forms, it is impossible to distinguish between MNAR and MAR types [19]. However, in the case of items missing from psychometric questionnaires (like the SF-36 in this study), an indirect approach can be used, based on the strong correlation between an item and its subscale (the SF-36 questionnaire was developed according to classical test theory to yield highly correlated items scale [12,13]): we therefore scored as "MNAR" those items for which the probability of missingness depended on, or was related to, the *score of subscale to which it belongs *(score computed without the missing item). We also used the socio-demographic and health status variables recorded in the 2003 Decennial Health Survey to distinguish between the MAR and MCAR types: if the probability of missingness for an item was found to depend on a predictor variable but not on its subscale score, the item non-response was classified as "MAR", whereas its was classified as "MCAR" if the probability of missingness depended neither on its subscale score nor on any (external) predictor variable.

Logistic regression models [20] were constructed to identify the type and determinants of missingness for each item of the SF-36 (except for HT). In these models, the dependent variable was binary: the item missing or not missing. The socio-demographic variables, those related to health status and those related to the SF-36 questionnaire were tested as predictor variables. The variables related to the SF-36 were the number of items of the questionnaire missing (in addition to the item analyzed) and the eight subscale scores, including the score for the scale to which the missing item belongs calculated without the missing item. All the variables tested, except the last which was selected to address the "MNAR hypothesis" (see above), addressed the "MAR hypothesis". Variables associated with the risk of item missingness in univariate analyses were used for multivariate analyses, and were entered into the final models using stepwise backward selection (remove p value = 0.05), modified to force gender and age into the models (because these variables have been already shown to be associated with the risk of missingness and could confound the association between missingness and many other predictors). The PROC LOGISTIC package of SAS software (v9.1, Cary, NC, USA) was used.

## Results

Table 1 summarizes the demographic and health characteristics of the survey participants. The missingness proportions for the 35 studied items of the SF-36 are given in Table 2. These proportions are not homogeneous, and fall between 2.4% (BP1) and 6.8% (GH5), with a mean of 4.4%.

**Table 1:** The 2003 Decennial Health Survey sample

	N	%	
**Socio-demographic data**			
**Age (Yrs)**			
19 - 29	3831	17	
30 - 39	4519	20	
40 - 49	4670	21	
50 - 59	4066	18	
60 - 69	2766	12	
70 - 79	2026	9	
> 80	742	3	
**Gender**			
Male	12123	46	
Female	10497	54	
**Education**			
no diploma	6392	28	
< high school graduate	8217	37	
high school graduate	5305	23	
university	2706	12	
**Occupation (present or past)**			
white collar	14194	64	
blue collar	6377	30	
no occupation	1467	6	
**French Nationality**			
yes	20810	92	
no	1810	8	
**Health status data**			
**Chronic disease**			
no	19798	88	
yes	2822	12	
**Hospitalization in the year**			
no	19580	87	
yes	3040	13	
**Vision disability**			
no	21658	96	
yes	962	4	
**Depression **(measured with the CES-D)			
no	16378	72	
yes	4694	21	
missing	1548	7	
**SF-36 questionnaire**			
**Number of missing items**			
0	16597	74	
1	1640	7	
2-3	2103	9	
≥ 4	2280	10	

**Subscales**	**median**	**mean**	**standard deviation**

PF: Physical Functioning	95	84	23
RP: Physical Role	100	81	33
BP: Bodily Pain	74	72	25
GH: Global Health	69	67	19
VT: Vitality	60	57	18
SF: Social Functioning	87	79	23
RE: Role emotional	100	81	34
MH: Mental Health	68	66	18

**Table 2:** Multivariate predictors of missingness for each item of the SF-36.

Scales/Items	Proportion of missing	Independent predictors	Type of missingness
**PF (Physical functioning)**			
PF1 Vigorous activities	3.1%	Age, Gender, Hospitalization, Number of missing data for other items	**MAR**
PF2 Moderate activities	3.2%	Age, Number of missing data for other items, PF score	**MNAR**
PF3 Lift, carry groceries	3.3%	Age, Number of missing data for other items, PF and GH scores	**MNAR**
PF4 Climb several flights	3.6%	Age, Occupation, Number of missing data for other items, PF and VT scores	**MNAR**
PF5 Climb one flight	4.9%	Age, Occupation, Education, Number of missing data for other items, PF and VT scores	**MNAR**
PF6 Bend, kneel	3.3%	Age, French nationality, Number of missing data for other items, PF score	**MNAR**
PF7 Walk>1 km	3.1%	Age, French nationality, Number of missing data for other items, PF score	**MNAR**
PF8 Walk several blocks	4.5%	Age, Number of missing data for other items, PF and SF scores	**MNAR**
PF9 Walk one block	2.8%	Chronic disease, Number of missing data for other items, PF score	**MNAR**
PF10 Bathe, dress	5.4%	Age, Number of missing data for other items, PF and VT scores	**MNAR**
**RP (Role limitations relating to physical health )**			
RP1 Cut down time on work	3.2%	Gender, Education, Number of missing data for other items, RE score	**MAR**
RP2 Accomplished less	3.2%	Number of missing data for other items, RP and GH scores	**MNAR**
RP3 Limited in kind of work	3.8%	Age, Number of missing data for other items, GH and RE scores	**MAR**
RP4 Difficulty performing work	3.5%	Age, French nationality, Number of missing data for other items, RP score	**MNAR**
**BP (Bodily pain)**			
BP1 Intensity of bodily pain	2.4%	Number of missing data for other items, PF and BP scores	**MNAR**
BP2 Extent pain interfered with work	2.7%	Number of missing data for other items	**MAR**
**GH (General health perceptions)**			
GH1 General health	6.4%	Age, Depression, Number of missing data for other items, SF score	**MAR**
GH2 Get sick easier	6.4%	Age, Number of missing data for other items, GH and SF scores	**MNAR**
GH3 As healthy as anybody	6.0%	Age, Hospitalization, Number of missing data for other items, GH score	**MNAR**
GH4 Expect health to get worse	6.1%	Age, Gender, French nationality, Number of missing data for other items	**MAR**
GH5 Health is excellent	6.8%	Age, Gender, Hospitalization, Number of missing data for other items, GH and SF scores	**MNAR**
**VT (Vitality)**			
VT1 Full of life	5.6%	Age, Education, Vision disability, Depression, Number of missing data for other items	**MAR**
VT2 Energy	5.6%	Age, Occupation, Number of missing data for other items	**MAR**
VT3 Worn out	5.5%	Age, Number of missing data for other items, BP score	**MAR**
VT4 Tired	4.0%	Number of missing data for other items	**MAR**
**SF (Social functioning)**			
SF1 Extent of social activities interfered with	2.6%	Gender, Number of missing data for other items, GH score	**MAR**
SF2 Frequency of social activities interfered with	3.0%	Age, Number of missing data for other items	**MAR**
**RE (Role limitation relating to mental health)**			
RE1 Cut down time on work	3.7%	Age, Number of missing data for other items, GH and RE scores	**MNAR**
RE2 Accomplished less	3.6%	Age, Number of missing data for other items, VT score	**MAR**
RE3 Did not do work as carefully	6.3%	Occupation, Number of missing data for other items, RE score	**MNAR**
**MH (Mental health)**			
MH1 Nervous	5.0%	Age, Number of missing data for other items, SF score	**MAR**
MH2 Down in the dumps	5.0%	Age, Number of missing data for other items	**MAR**
MH3 Peaceful	5.3%	Education, Vision disability, Number of missing data for other items	**MAR**
MH4 Blue/sad	5.2%	Gender, Depression, Number of missing data for other items, VT scale	**MAR**
MH5 Happy	5.2%	Age, Gender, Number of missing data for other items, GH scale	**MAR**

Multivariate predictors of missingness are presented in Table 2 (the detailed results of the univariate and multivariate analyses are given in Additional files 2 and 3). For the items PF1, RP1, RP3, BP2, GH1, GH4, RE2 and the items of the subscales VT, SF and MH, only "external" determinants were found and they can therefore be classified as missing at random (MAR). Missingness for all other items depended on their subscale score and can therefore be classified as missing not at random (MNAR).

Age had a strong and similar effect on missingness for almost all items, with an increase in the proportion of missing data of 10 to 50% per 10 years of age. Data was more frequently missing for women than men for most items but the difference was less systematic than that observed between age groups. Nevertheless, for some items (RP1, SF1), the risk of missingness was twice as high, or higher, for women than men. Other socio-demographic variables (educational level, occupation, nationality) were also significantly correlated with the risk of missingness: the proportion of missing data for PF5, RP1, VT1, MH3 increased with decreasing educational level. Similarly, missing data was more frequent for PF4, PF5, VT2 and RE3 for "blue collar workers" than other groups and for PF6, PF7, RP4 and GH4 for non-national than French subjects.

Missingness increased only for some items with poorer health status: subjects having been hospitalized in the year had higher proportion of missing data for PF1, GH3 and GH5; those with chronic disease(s) for PF9; and subjects with depression as classified by the CES-D for GH1, VT1 and MH4. Subjects with vision problems had higher proportion of missing data for and VT1 and MH3.

Low scores on the SF-36 subscales predicted missingness for more than half of the items belonging to their scales (indicating a "MNAR" process, see above). However, there were some more diffuse or "collateral" effects on items belonging to different sub-scales. For example, a low RE subscale score increased the risk of missingness for RE1 and RE3 (MNAR items) and also for RP1 and RP3; a low VT score increased the risk of missingness for PF4, PF5, PF10, RE2 and MH4. The atypical findings for the item BP1 are interesting: for this item ("How much bodily pain...") both univariate and multivariate analyses revealed that the proportion of missing data increased with increasing score on the BP subscale i.e. with decreasing perceived pain. The number of missing items was predictive of missingness for all items, with the OR range being from 1.42 (for BP1) to 2.65 (for PF8).

## Discussion

We exploited the French 2003 Decennial Health Survey to investigate diverse socio-demographic, health status and QoL variables as potential predictors of item missingness in the SF-36 questionnaire; we also used the classification proposed by Little and Rubin to characterize missing data processes operating during administration of this questionnaire. In this large representative sample of the French population the proportion of missing items varied between 2% and 7%. The type of missingness was missing at random for 18 items (items PF1, RP1, RP3, BP2, GH1, GH4, RE2 and all items of VT, SF and MH subscales) and missing not at random for the others (items PF2-10, RP2, RP4, BP1, GH2, GH3, GH5, RE1 and RE3). No item was missing completely at random (MCAR). MCAR is the only "ignorable" missing data process [17], so our results imply that it is necessary to use an imputation technique to correct for biases associated with missing values when using the SF-36. The personal mean score, where the imputed value of a missing item is the mean of the non-missing items of the same scale, has been recommended for use with the SF-36 [15,16]. Other imputation methods, notably the hot deck [21] and multiple imputation [22,23], have been gaining popularity in clinical and epidemiological research and have been considered for use in QoL research [4,5]; they may be applicable to the SF-36 (these techniques are being compared and the results will be reported elsewhere -- manuscript in preparation).

However, prevention is undoubtedly the optimal approach to the issue of missing data [24]. Consequently, it is important to identify the factors associated with the occurrence of missing data as this could help prevention. Our results confirm the earlier findings of Perneger and Burnand with the SF-12 [4] and of Vercherin et al. with the SF-36 [8], that older age, female sex, and to a lesser extent low education and low economic status (blue collar workers and non-nationals), are major determinants of item missingness in QoL questionnaires. Although some of these questionnaires have been carefully constructed and tested to be administered to large populations (as was the SF-36), it appears that some questions may be too difficult to understand for some subjects (low educational level, foreigners) and that others (seemingly more numerous) may be perceived as being of no interest or even inappropriate for women and particularly older members of the population. Subjects with deteriorated health status and those with altered QoL were also found to be independently (and independently of other characteristics) prone to respond with missing items. It is likely that these individuals may tend to avoid questions which are embarrassing or cause distress [3].

Finally, the present study has various limitations that need to be considered. The only moderate fit of some final models indicates that not all the predictors of missingness were identified. An additional limitation is that only an indirect approach could be used to identify the MNAR process. However, direct identification would have required contacting all the subjects to ask them to fully fill in the missing items (which was clearly impossible in this large population-based study).

## Conclusion

In conclusion, our analysis shows that imputation of missing items in the responses to the SF-36 questionnaire is necessary and identifies several factors that should be carefully considered when designing strategies for the prevention of missing data in the SF-36. Methodologies similar to that we describe here could be used to address the issue of item missingness in other QoL questionnaires.

## Abbreviations

MCAR: Missing completely at random; MAR: Missing At Random; MNAR: Missing Not At Random; QoL: Quality of life; SF-36: Medical Outcome Study 36-item short-form health survey.

## Competing interests

The authors declare that they have no competing interests.

## Authors' contributions

HP participated in the design of the study, performed the statistical analysis and drafted the manuscript. JC and AL conceived the study, participated in its design and helped to draft the manuscript. JC provided administrative, technical and logistic support. All authors read and approved the final manuscript.

## Supplementary Material

Additional file 1 Scales, items of the SF-36 questionnaire and their scores. Click here for file

Additional file 2 Univariate analysis for factors associated with the missingness for each item of the SF-36. Click here for file

Additional file 3 Multivariate analysis for factors associated with the missingness for each item of the SF-36. Click here for file
