# Supplementary material for: Identifying type and determinants of missing items in quality of life questionnaires: Application to the SF-36 French version of the 2003 Decennial Health Survey
Source: Health Qual Life Outcomes. 2010 Feb 3;8:16. doi: 10.1186/1477-7525-8-16 (PMC2841108; doi:10.1186/1477-7525-8-16)
Supplement: Additional file 1 — Scales, items of the SF-36 questionnaire and their scores. [file 1477-7525-8-16-S1.DOC]

**Additional file 1**: Scales, items of the SF-36 questionnaire and their scores.

| **Scales** / Items | **Scores (ordinal Likert)** |
| --- | --- |
| **PF (Physical functioning )** |  |
| PF1 Vigorous activities | 1 to 3 |
| PF2 Moderate activities | 1 to 3 |
| PF3 Lift, carry groceries | 1 to 3 |
| PF4 Climb several flights | 1 to 3 |
| PF5 Climb one flight | 1 to 3 |
| PF6 Bend, kneel | 1 to 3 |
| PF7 Walk>1 km | 1 to 3 |
| PF8 Walk several blocks | 1 to 3 |
| PF9 Walk one block | 1 to 3 |
| PF10 Bathe, dress | 1 to 3 |
| **RP (Role limitations relating to physical health )** |  |
| RP1 Cut down time on work | 1 to 2 |
| RP2 Accomplished less | 1 to 2 |
| RP3 Limited in kind of work | 1 to 2 |
| RP4 Difficulty performing work | 1 to 2 |
| **BP (Bodily pain)** |  |
| BP1 Intensity of bodily pain | 1 to 6 |
| BP2 Extent pain interfered with work | 1 to 6 |
| **GH (General health perceptions)** |  |
| GH1 General health | 1 to 5 |
| GH2 Get sick easier | 1 to 5 |
| GH3 As healthy as anybody | 1 to 5 |
| GH4 Expect health to get worse | 1 to 5 |
| GH5 Health is excellent | 1 to 5 |
| **VT (Vitality)** |  |
| VT1 Full of life | 1 to 6 |
| VT2 Energy | 1 to 6 |
| VT3 Worn out | 1 to 6 |
| VT4 Tired | 1 to 6 |
| **SF (Social functioning)** |  |
| SF1 Extent of social activities interfered with | 1 to 5 |
| SF2 Frequency of social activities interfered with | 1 to 5 |
| **RE (Role limitation relating to mental health)** |  |
| RE1 Cut down time on work | 1 to 2 |
| RE2 Accomplished less | 1 to 2 |
| RE3 Did not do work as carefully | 1 to 2 |
| **MH (Mental health)** |  |
| MH1 Nervous | 1 to 6 |
| MH2 Down in the dumps | 1 to 6 |
| MH3 Peaceful | 1 to 6 |
| MH4 Blue/sad | 1 to 6 |
| MH5 Happy | 1 to 6 |
|  |  |
| HT (Transition)  Health compared to 1 year ago (one item) | 1 to 5 |
