# Supplementary material for: Identifying type and determinants of missing items in quality of life questionnaires: Application to the SF-36 French version of the 2003 Decennial Health Survey
Source: Health Qual Life Outcomes. 2010 Feb 3;8:16. doi: 10.1186/1477-7525-8-16 (PMC2841108; doi:10.1186/1477-7525-8-16)
Supplement: Additional file 2 — Univariate analysis for factors associated with the missingness for each item of the SF-36. [file 1477-7525-8-16-S2.DOC]

**Additional file 2:** Univariate analysis for factors associated with the missingness for each item of the SF-36.

|  |  |  | **PF1** | | | | |  |  | **PF2** | | | | |  |  | **PF3** | | | | |  |  | **PF4** | | | | |  |  | **PF5** | | | | |  |  | **PF6** | | | | |  |  | **PF7** | | | | |  |  | **PF8** | | | | |  |  | **PF9** | | | |
| --- | --- | --- | --- | --- | --- | --- | --- | --- | --- | --- | --- | --- | --- | --- | --- | --- | --- | --- | --- | --- | --- | --- | --- | --- | --- | --- | --- | --- | --- | --- | --- | --- | --- | --- | --- | --- | --- | --- | --- | --- | --- | --- | --- | --- | --- | --- | --- | --- | --- | --- | --- | --- | --- | --- | --- | --- | --- | --- | --- | --- | --- | --- |
|  | **Proportion of missing data** | | **3.1%** | | | | |  |  | **3.2%** | | | | |  |  | **3.3%** | | | | |  |  | **3.6%** | | | | |  |  | **4.9%** | | | | |  |  | **3.3%** | | | | |  |  | **3.1%** | | | | |  |  | **4.5%** | | | | |  |  | **2.8%** | | | |
|  |  |  | **OR** |  | **(95% CI)** | | |  |  | **OR** |  | **(95% CI)** | | |  |  | **OR** |  | **(95% CI)** | | |  |  | **OR** |  | **(95% CI)** | | |  |  | **OR** |  | **(95% CI)** | | |  |  | **OR** |  | **(95% CI)** | | |  |  | **OR** |  | **(95% CI)** | | |  |  | **OR** |  | **(95% CI)** | | |  |  | **OR** |  | **(95% CI)** | |
|  |  |  |  |  |  |  |  |  |  |  |  |  |  |  |  |  |  |  |  |  |  |  |  |  |  |  |  |  |  |  |  |  |  |  |  |  |  |  |  |  |  |  |  |  |  |  |  |  |  |  |  |  |  |  |  |  |  |  |  |  |  |  |
|  | **Age**¹ |  | **1.63** |  | (1.55 | - | 1.70) |  |  | **1.64** |  | (1.56 | - | 1.71) |  |  | **1.65** |  | (1.57 | - | 1.72) |  |  | **1.70** |  | (1.62 | - | 1.77) |  |  | **1.65** |  | (1.59 | - | 1.71) |  |  | **1.58** |  | (1.51 | - | 1.65) |  |  | **1.63** |  | (1.55 | - | 1.70) |  |  | **1.73** |  | (1.66 | - | 1.80) |  |  | **1.55** |  | (1.48 | -1.62 ) |
|  | **Gender** | Male |  |  |  |  |  |  |  |  |  |  |  |  |  |  |  |  |  |  |  |  |  |  |  |  |  |  |  |  |  |  |  |  |  |  |  |  |  |  |  |  |  |  |  |  |  |  |  |  |  |  |  |  |  |  |  |  |  |  |  |  |
|  |  | Female | **1.52** |  | (1.30 | - | 1.78) |  |  | 1.06 |  | (0.91 | - | 1.23) |  |  | 1.05 |  | (0.90 | - | 1.21) |  |  | **1.22** |  | (1.05 | - | 1.40) |  |  | **1.18** |  | (1.04 | - | 1.34) |  |  | 1.15 |  | (0.99 | - | 1.33) |  |  | **1.24** |  | (1.07 | - | 1.45) |  |  | 1.11 |  | (0.98 | - | 1.26) |  |  | 1.03 |  | (0.88 | -1.21) |
|  | **Education** | no diploma |  |  |  |  |  |  |  |  |  |  |  |  |  |  |  |  |  |  |  |  |  |  |  |  |  |  |  |  |  |  |  |  |  |  |  |  |  |  |  |  |  |  |  |  |  |  |  |  |  |  |  |  |  |  |  |  |  |  |  |  |
|  |  | < HS graduate | **0.30** |  | (0.25 | - | 0.36) |  |  | **0.25** |  | (0.21 | - | 0.30) |  |  | **0.27** |  | (0.22 | - | 0.32) |  |  | **0.27** |  | (0.22 | - | 0.32) |  |  | **0.27** |  | (0.23 | - | 0.31) |  |  | **0.26** |  | (0.22 | - | 0.31) |  |  | **0.26** |  | (0.22 | - | 0.32) |  |  | **0.26** |  | (0.22 | - | 0.30) |  |  | **0.24** |  | (0.20 | -0.29) |
|  |  | HS graduate | **0.23** |  | (0.18 | - | 0.29) |  |  | **0.18** |  | (0.14 | - | 0.23) |  |  | **0.20** |  | (0.15 | - | 0.25) |  |  | **0.19** |  | (0.15 | - | 0.24) |  |  | **0.19** |  | (0.15 | - | 0.23) |  |  | **0.21** |  | (0.17 | - | 0.27) |  |  | **0.20** |  | (0.16 | - | 0.26) |  |  | **0.19** |  | (0.15 | - | 0.23) |  |  | **0.18** |  | (0.14 | -0.23) |
|  |  | university | **0.14** |  | (0.09 | - | 0.21) |  |  | **0.12** |  | (0.08 | - | 0.18) |  |  | **0.13** |  | (0.09 | - | 0.19) |  |  | **0.11** |  | (0.07 | - | 0.16) |  |  | **0.14** |  | (0.10 | - | 0.19) |  |  | **0.16** |  | (0.12 | - | 0.23) |  |  | **0.14** |  | (0.09 | - | 0.20) |  |  | **0.15** |  | (0.11 | - | 0.21) |  |  | **0.10** |  | (0.07 | -0.17) |
|  | **Occupation**² | white collar |  |  |  |  |  |  |  |  |  |  |  |  |  |  |  |  |  |  |  |  |  |  |  |  |  |  |  |  |  |  |  |  |  |  |  |  |  |  |  |  |  |  |  |  |  |  |  |  |  |  |  |  |  |  |  |  |  |  |  |  |
|  |  | blue collar | **1.58** |  | (1.34 | - | 1.86) |  |  | **2.30** |  | (1.97 | - | 2.69) |  |  | **2.10** |  | (1.80 | - | 2.45) |  |  | **2.18** |  | (1.88 | - | 2.52) |  |  | **1.94** |  | (1.70 | - | 2.20) |  |  | **2.05** |  | (1.76 | - | 2.39) |  |  | **2.12** |  | (1.81 | - | 2.48) |  |  | **1.85** |  | (1.62 | - | 2.11) |  |  | **2.39** |  | (2.03 | -2.83) |
|  | **French nationality** | yes |  |  |  |  |  |  |  |  |  |  |  |  |  |  |  |  |  |  |  |  |  |  |  |  |  |  |  |  |  |  |  |  |  |  |  |  |  |  |  |  |  |  |  |  |  |  |  |  |  |  |  |  |  |  |  |  |  |  |  |  |
|  |  | no | **1.37** |  | (1.07 | - | 1.75) |  |  | 1.26 |  | (0.98 | - | 1.62) |  |  | 1.05 |  | (0.81 | - | 1.37) |  |  | **1.31** |  | (1.04 | - | 1.66) |  |  | **1.23** |  | (1.00 | - | 1.52) |  |  | **1.60** |  | (1.28 | - | 2.01) |  |  | **1.46** |  | (1.15 | - | 1.86) |  |  | **1.39** |  | (1.13 | - | 1.71) |  |  | **1.54** |  | (1.20 | -1.98) |
|  | **Chronic disease** | no |  |  |  |  |  |  |  |  |  |  |  |  |  |  |  |  |  |  |  |  |  |  |  |  |  |  |  |  |  |  |  |  |  |  |  |  |  |  |  |  |  |  |  |  |  |  |  |  |  |  |  |  |  |  |  |  |  |  |  |  |
|  |  | yes | **2.01** |  | (1.67 | - | 2.42) |  |  | **2.43** |  | (2.04 | - | 2.89) |  |  | **2.03** |  | (1.69 | - | 2.42) |  |  | **2.04** |  | (1.72 | - | 2.43) |  |  | **2.05** |  | (1.77 | - | 2.38) |  |  | **2.11** |  | (1.77 | - | 2.52) |  |  | **2.03** |  | (1.69 | - | 2.44) |  |  | **2.24** |  | (1.93 | - | 2.61) |  |  | **2.21** |  | (1.83 | -2.67) |
|  | **Hospitalization** | no |  |  |  |  |  |  |  |  |  |  |  |  |  |  |  |  |  |  |  |  |  |  |  |  |  |  |  |  |  |  |  |  |  |  |  |  |  |  |  |  |  |  |  |  |  |  |  |  |  |  |  |  |  |  |  |  |  |  |  |  |
|  | **in the year** | yes | **1.32** |  | (1.08 | - | 1.62) |  |  | 1.13 |  | (0.91 | - | 1.39) |  |  | 1.20 |  | (0.98 | - | 1.47) |  |  | **1.22** |  | (1.01 | - | 1.49) |  |  | **1.24** |  | (1.05 | - | 1.47) |  |  | **1.32** |  | (1.09 | - | 1.61) |  |  | **1.32** |  | (1.08 | - | 1.62) |  |  | **1.24** |  | (1.05 | - | 1.48) |  |  | **1.26** |  | (1.02 | -1.57) |
|  | **Vision disability** | no |  |  |  |  |  |  |  |  |  |  |  |  |  |  |  |  |  |  |  |  |  |  |  |  |  |  |  |  |  |  |  |  |  |  |  |  |  |  |  |  |  |  |  |  |  |  |  |  |  |  |  |  |  |  |  |  |  |  |  |  |
|  |  | yes | 1.17 |  | (0.82 | - | 1.66) |  |  | 1.17 |  | (0.83 | - | 1.66) |  |  | 1.19 |  | (0.85 | - | 1.68) |  |  | 1.09 |  | (0.78 | - | 1.53) |  |  | 1.27 |  | (0.97 | - | 1.67) |  |  | 1.15 |  | (0.82 | - | 1.62) |  |  | 1.31 |  | (0.94 | - | 1.83) |  |  | 1.12 |  | (0.83 | - | 1.53) |  |  | 1.22 |  | (0.85 | -1.76) |
|  | **Depression**³ | no |  |  |  |  |  |  |  |  |  |  |  |  |  |  |  |  |  |  |  |  |  |  |  |  |  |  |  |  |  |  |  |  |  |  |  |  |  |  |  |  |  |  |  |  |  |  |  |  |  |  |  |  |  |  |  |  |  |  |  |  |
|  |  | yes | **1.63** |  | (1.34 | - | 1.98) |  |  | **1.76** |  | (1.44 | - | 2.14) |  |  | **1.72** |  | (1.42 | - | 2.08) |  |  | **1.60** |  | (1.33 | - | 1.93) |  |  | **1.68** |  | (1.44 | - | 1.96) |  |  | **1.74** |  | (1.44 | - | 2.11) |  |  | **1.64** |  | (1.34 | - | 2.00) |  |  | **1.62** |  | (1.38 | - | 1.90) |  |  | **1.73** |  | (1.40 | -2.13) |
|  | **Number of missing data  for other items** | | **1.22** |  | (1.21 | - | 1.23) |  |  | **1.29** |  | (1.27 | - | 1.30) |  |  | **1.28** |  | (1.26 | - | 1.29) |  |  | **1.27** |  | (1.26 | - | 1.29) |  |  | **1.24** |  | (1.23 | - | 1.25) |  |  | **1.27** |  | (1.26 | - | 1.29) |  |  | **1.29** |  | (1.27 | - | 1.30) |  |  | **1.29** |  | (1.28 | - | 1.30) |  |  | **1.30** |  | (1.29 | -1.32) |
|  | **Subscales**^4^ | PF | **0.78** |  | (0.76 | - | 0.80) |  |  | **0.71** |  | (0.69 | - | 0.73) |  |  | **0.72** |  | (0.71 | - | 0.74) |  |  | **0.73** |  | (0.71 | - | 0.75) |  |  | **0.74** |  | (0.73 | - | 0.76) |  |  | **0.73** |  | (0.71 | - | 0.75) |  |  | **0.71** |  | (0.69 | - | 0.72) |  |  | **0.73** |  | (0.72 | - | 0.75) |  |  | **0.70** |  | (0.68 | -0.72) |
|  |  | RP | **0.93** |  | (0.91 | - | 0.95) |  |  | **0.88** |  | (0.85 | - | 0.90) |  |  | **0.87** |  | (0.85 | - | 0.89) |  |  | **0.88** |  | (0.86 | - | 0.90) |  |  | **0.89** |  | (0.87 | - | 0.90) |  |  | **0.88** |  | (0.86 | - | 0.90) |  |  | **0.87** |  | (0.85 | - | 0.89) |  |  | **0.89** |  | (0.87 | - | 0.91) |  |  | **0.87** |  | (0.84 | -0.89) |
|  |  | BP | **0.92** |  | (0.89 | - | 0.95) |  |  | **0.89** |  | (0.87 | - | 0.92) |  |  | **0.89** |  | (0.86 | - | 0.92) |  |  | **0.89** |  | (0.87 | - | 0.92) |  |  | **0.89** |  | (0.87 | - | 0.91) |  |  | **0.90** |  | (0.87 | - | 0.92) |  |  | **0.88** |  | (0.86 | - | 0.91) |  |  | **0.91** |  | (0.88 | - | 0.93) |  |  | **0.89** |  | (0.86 | -0.92) |
|  |  | GH | **0.88** |  | (0.84 | - | 0.93) |  |  | **0.85** |  | (0.81 | - | 0.89) |  |  | **0.83** |  | (0.79 | - | 0.87) |  |  | **0.87** |  | (0.83 | - | 0.91) |  |  | **0.85** |  | (0.82 | - | 0.88) |  |  | **0.85** |  | (0.81 | - | 0.89) |  |  | **0.84** |  | (0.80 | - | 0.88) |  |  | **0.88** |  | (0.84 | - | 0.91) |  |  | **0.83** |  | (0.79 | -0.87) |
|  |  | VT | **0.92** |  | (0.87 | - | 0.96) |  |  | **0.88** |  | (0.84 | - | 0.93) |  |  | **0.88** |  | (0.83 | - | 0.92) |  |  | **0.92** |  | (0.87 | - | 0.96) |  |  | **0.89** |  | (0.86 | - | 0.93) |  |  | **0.88** |  | (0.84 | - | 0.92) |  |  | **0.86** |  | (0.81 | - | 0.90) |  |  | **0.92** |  | (0.88 | - | 0.96) |  |  | **0.88** |  | (0.83 | -0.93) |
|  |  | SF | **0.88** |  | (0.85 | - | 0.90) |  |  | **0.83** |  | (0.81 | - | 0.85) |  |  | **0.83** |  | (0.81 | - | 0.86) |  |  | **0.85** |  | (0.82 | - | 0.87) |  |  | **0.84** |  | (0.82 | - | 0.86) |  |  | **0.83** |  | (0.81 | - | 0.85) |  |  | **0.82** |  | (0.80 | - | 0.85) |  |  | **0.86** |  | (0.83 | - | 0.88) |  |  | **0.81** |  | (0.79 | -0.84) |
|  |  | RE | **0.94** |  | (0.91 | - | 0.96) |  |  | **0.90** |  | (0.87 | - | 0.92) |  |  | **0.89** |  | (0.87 | - | 0.92) |  |  | **0.90** |  | (0.88 | - | 0.93) |  |  | **0.91** |  | (0.89 | - | 0.93) |  |  | **0.90** |  | (0.88 | - | 0.92) |  |  | **0.89** |  | (0.87 | - | 0.91) |  |  | **0.91** |  | (0.90 | - | 0.93) |  |  | **0.90** |  | (0.88 | -0.93) |
|  |  | MH | **0.92** |  | (0.87 | - | 0.96) |  |  | **0.88** |  | (0.83 | - | 0.93) |  |  | **0.88** |  | (0.83 | - | 0.92) |  |  | **0.92** |  | (0.87 | - | 0.96) |  |  | **0.89** |  | (0.86 | - | 0.93) |  |  | **0.88** |  | (0.83 | - | 0.92) |  |  | **0.88** |  | (0.84 | - | 0.93) |  |  | **0.93** |  | (0.89 | - | 0.97) |  |  | **0.87** |  | (0.82 | -0.93) |
|  |  |  |  |  |  |  |  |  |  |  |  |  |  |  |  |  |  |  |  |  |  |  |  |  |  |  |  |  |  |  |  |  |  |  |  |  |  |  |  |  |  |  |  |  |  |  |  |  |  |  |  |  |  |  |  |  |  |  |  |  |  |  |

OR = odds ratios; 95 %CI: 95% Confidence interval

¹ OR associated with an increment of 10 years

² Subjects without an occupation are excluded from this analysis

³ Depression as measured by the CES-D; CES-D depression score is the sum of the 20 items, further dichotomized (a score ≥ 16 indicates depression; the score is not computed if more than 4 items are missing).

^4^ OR associated with an increment of 10 points for all subscale scores

**Additional file 2:** Continued

|  |  | **PF10** | | | | |  |  | **RP1** | | | | |  |  | **RP2** | | | | |  |  | **RP3** | | | | |  |  | **RP4** | | | | |  |  | **BP1** | | | | |  |  | **BP2** | | | | |  |  | **GH1** | | | | |  |  | **GH2** | | | |  |
| --- | --- | --- | --- | --- | --- | --- | --- | --- | --- | --- | --- | --- | --- | --- | --- | --- | --- | --- | --- | --- | --- | --- | --- | --- | --- | --- | --- | --- | --- | --- | --- | --- | --- | --- | --- | --- | --- | --- | --- | --- | --- | --- | --- | --- | --- | --- | --- | --- | --- | --- | --- | --- | --- | --- | --- | --- | --- | --- | --- | --- | --- | --- |
|  |  |  |  |  |  |  |  |  |  |  |  |  |  |  |  |  |  |  |  |  |  |  |  |  |  |  |  |  |  |  |  |  |  |  |  |  |  |  |  |  |  |  |  |  |  |  |  |  |  |  |  |  |  |  |  |  |  |  |  |  |  |  |
| **Proportion of missing data** | | **5.4%** | | | | |  |  | **3.2%** | | | | |  |  | **3.2%** | | | | |  |  | **3.8%** | | | | |  |  | **3.5%** | | | | |  |  | **2.4%** | | | | |  |  | **2.7%** | | | | |  |  | **6.4%** | | | | |  |  | **6.4%** | | | | |
|  |  | **OR** |  | **(95% CI)** | | |  |  | **OR** |  | **(95% CI)** | | |  |  | **OR** |  | **(95% CI)** | | |  |  | **OR** |  | **(95% CI)** | | |  |  | **OR** |  | **(95% CI)** | | |  |  | **OR** |  | **(95% CI)** | | |  |  | **OR** |  | **(95% CI)** | | |  |  | **OR** |  | **(95% CI)** | | |  |  | **OR** |  | **(95% CI)** | | |
|  |  |  |  |  |  |  |  |  |  |  |  |  |  |  |  |  |  |  |  |  |  |  |  |  |  |  |  |  |  |  |  |  |  |  |  |  |  |  |  |  |  |  |  |  |  |  |  |  |  |  |  |  |  |  |  |  |  |  |  |  |  |  |
| **Age**¹ |  | **1.75** |  | (1.69 | - | 1.82) |  |  | **1.74** |  | (1.66 | - | 1.82) |  |  | **1.66** |  | (1.59 | - | 1.74) |  |  | **1.63** |  | (1.56 | - | 1.70) |  |  | **1.66** |  | (1.59 | - | 1.73) |  |  | **1.34** |  | (1.27 | - | 1.40) |  |  | **1.40** |  | (1.33 | - | 1.47) |  |  | **1.06** |  | (1.03 | - | 1.10) |  |  | **1.63** |  | (1.58 | - | 1.69) |
| Gender | Male |  |  |  |  |  |  |  |  |  |  |  |  |  |  |  |  |  |  |  |  |  |  |  |  |  |  |  |  |  |  |  |  |  |  |  |  |  |  |  |  |  |  |  |  |  |  |  |  |  |  |  |  |  |  |  |  |  |  |  |  |  |
|  | Female | **1.15** |  | (1.03 | - | 1.30) |  |  | **1.44** |  | (1.23 | - | 1.67) |  |  | **1.30** |  | (1.12 | - | 1.51) |  |  | **1.31** |  | (1.14 | - | 1.50) |  |  | **1.27** |  | (1.10 | - | 1.47) |  |  | **1.26** |  | (1.06 | - | 1.50) |  |  | **1.25** |  | (1.06 | - | 1.47) |  |  | **1.18** |  | (1.06 | - | 1.33) |  |  | **1.21** |  | (1.09 | - | 1.35) |
| **Education** | no diploma |  |  |  |  |  |  |  |  |  |  |  |  |  |  |  |  |  |  |  |  |  |  |  |  |  |  |  |  |  |  |  |  |  |  |  |  |  |  |  |  |  |  |  |  |  |  |  |  |  |  |  |  |  |  |  |  |  |  |  |  |  |
|  | < HS graduate | **0.25** |  | (0.22 | - | 0.29) |  |  | **0.22** |  | (0.18 | - | 0.27) |  |  | **0.23** |  | (0.19 | - | 0.27) |  |  | **0.26** |  | (0.22 | - | 0.30) |  |  | **0.24** |  | (0.20 | - | 0.29) |  |  | **0.46** |  | (0.38 | - | 0.56) |  |  | **0.45** |  | (0.37 | - | 0.54) |  |  | 1.03 |  | (0.90 | - | 1.17) |  |  | **0.35** |  | (0.31 | - | 0.40) |
|  | HS graduate | **0.19** |  | (0.15 | - | 0.22) |  |  | **0.18** |  | (0.14 | - | 0.23) |  |  | **0.23** |  | (0.18 | - | 0.29) |  |  | **0.22** |  | (0.18 | - | 0.27) |  |  | **0.23** |  | (0.19 | - | 0.29) |  |  | **0.35** |  | (0.27 | - | 0.45) |  |  | **0.37** |  | (0.29 | - | 0.46) |  |  | 0.88 |  | (0.76 | - | 1.02) |  |  | **0.25** |  | (0.21 | - | 0.29) |
|  | university | **0.15** |  | (0.11 | - | 0.20) |  |  | **0.07** |  | (0.04 | - | 0.12) |  |  | **0.11** |  | (0.07 | - | 0.16) |  |  | **0.13** |  | (0.09 | - | 0.18) |  |  | **0.12** |  | (0.08 | - | 0.17) |  |  | **0.25** |  | (0.17 | - | 0.36) |  |  | **0.24** |  | (0.16 | - | 0.35) |  |  | **0.70** |  | (0.57 | - | 0.85) |  |  | **0.20** |  | (0.16 | - | 0.26) |
| **Occupation**² | white collar |  |  |  |  |  |  |  |  |  |  |  |  |  |  |  |  |  |  |  |  |  |  |  |  |  |  |  |  |  |  |  |  |  |  |  |  |  |  |  |  |  |  |  |  |  |  |  |  |  |  |  |  |  |  |  |  |  |  |  |  |  |
|  | blue collar | **1.78** |  | (1.57 | - | 2.01) |  |  | **2.00** |  | (1.71 | - | 2.34) |  |  | **2.04** |  | (1.75 | - | 2.38) |  |  | **1.99** |  | (1.72 | - | 2.29) |  |  | **1.99** |  | (1.72 | - | 2.31) |  |  | **1.51** |  | (1.25 | - | 1.81) |  |  | **1.53** |  | (1.28 | - | 1.82) |  |  | 1.03 |  | (0.92 | - | 1.16) |  |  | **1.60** |  | (1.43 | - | 1.80) |
| **French nationality** | yes |  |  |  |  |  |  |  |  |  |  |  |  |  |  |  |  |  |  |  |  |  |  |  |  |  |  |  |  |  |  |  |  |  |  |  |  |  |  |  |  |  |  |  |  |  |  |  |  |  |  |  |  |  |  |  |  |  |  |  |  |  |
|  | no | **1.42** |  | (1.17 | - | 1.71) |  |  | 1.23 |  | (0.96 | - | 1.59) |  |  | **1.39** |  | (1.09 | - | 1.77) |  |  | **1.29** |  | (1.03 | - | 1.62) |  |  | **1.34** |  | (1.06 | - | 1.70) |  |  | 1.11 |  | (0.82 | - | 1.50) |  |  | 1.16 |  | (0.86 | - | 1.54) |  |  | 1.10 |  | (0.91 | - | 1.33) |  |  | 1.17 |  | (0.98 | - | 1.41) |
| **Chronic disease** | no |  |  |  |  |  |  |  |  |  |  |  |  |  |  |  |  |  |  |  |  |  |  |  |  |  |  |  |  |  |  |  |  |  |  |  |  |  |  |  |  |  |  |  |  |  |  |  |  |  |  |  |  |  |  |  |  |  |  |  |  |  |
|  | yes | **2.32** |  | (2.02 | - | 2.66) |  |  | **3.00** |  | (2.54 | - | 3.54) |  |  | **2.79** |  | (2.36 | - | 3.31) |  |  | **2.52** |  | (2.15 | - | 2.95) |  |  | **2.71** |  | (2.30 | - | 3.19) |  |  | **1.55** |  | (1.24 | - | 1.94) |  |  | **1.78** |  | (1.45 | - | 2.19) |  |  | **1.22** |  | (1.05 | - | 1.42) |  |  | **2.18** |  | (1.91 | - | 2.49) |
| **Hospitalization** | no |  |  |  |  |  |  |  |  |  |  |  |  |  |  |  |  |  |  |  |  |  |  |  |  |  |  |  |  |  |  |  |  |  |  |  |  |  |  |  |  |  |  |  |  |  |  |  |  |  |  |  |  |  |  |  |  |  |  |  |  |  |
| **in the year** | yes | **1.22** |  | (1.04 | - | 1.43) |  |  | **1.53** |  | (1.27 | - | 1.86) |  |  | **1.48** |  | (1.22 | - | 1.80) |  |  | **1.34** |  | (1.11 | - | 1.61) |  |  | **1.58** |  | (1.32 | - | 1.89) |  |  | 1.25 |  | (0.99 | - | 1.58) |  |  | 1.20 |  | (0.96 | - | 1.51) |  |  | 1.05 |  | (0.90 | - | 1.23) |  |  | **1.55** |  | (1.35 | - | 1.79) |
| **Vision disability** | no |  |  |  |  |  |  |  |  |  |  |  |  |  |  |  |  |  |  |  |  |  |  |  |  |  |  |  |  |  |  |  |  |  |  |  |  |  |  |  |  |  |  |  |  |  |  |  |  |  |  |  |  |  |  |  |  |  |  |  |  |  |
|  | yes | 1.10 |  | (0.83 | - | 1.45) |  |  | **1.38** |  | (1.00 | - | 1.90) |  |  | 1.12 |  | (0.79 | - | 1.60) |  |  | 1.13 |  | (0.82 | - | 1.56) |  |  | 1.02 |  | (0.72 | - | 1.45) |  |  | 1.01 |  | (0.66 | - | 1.54) |  |  | 1.03 |  | (0.69 | - | 1.54) |  |  | 0.92 |  | (0.70 | - | 1.22) |  |  | 1.20 |  | (0.94 | - | 1.54) |
| **Depression**³ | no |  |  |  |  |  |  |  |  |  |  |  |  |  |  |  |  |  |  |  |  |  |  |  |  |  |  |  |  |  |  |  |  |  |  |  |  |  |  |  |  |  |  |  |  |  |  |  |  |  |  |  |  |  |  |  |  |  |  |  |  |  |
|  | yes | **1.65** |  | (1.43 | - | 1.92) |  |  | **2.63** |  | (2.17 | - | 3.19) |  |  | **1.98** |  | (1.62 | - | 2.41) |  |  | **1.93** |  | (1.62 | - | 2.31) |  |  | **1.82** |  | (1.50 | - | 2.19) |  |  | **1.47** |  | (1.14 | - | 1.88) |  |  | **1.45** |  | (1.14 | - | 1.84) |  |  | **1.30** |  | (1.14 | - | 1.47) |  |  | **1.64** |  | (1.43 | - | 1.89) |
| **Number of missing data  for other items** | | **1.29** |  | (1.27 | - | 1.30) |  |  | **1.26** |  | (1.25 | - | 1.28) |  |  | **1.29** |  | (1.28 | - | 1.31) |  |  | **1.27** |  | (1.25 | - | 1.28) |  |  | **1.28** |  | (1.27 | - | 1.29) |  |  | **1.24** |  | (1.23 | - | 1.26) |  |  | **1.25** |  | (1.24 | - | 1.26) |  |  | **1.07** |  | (1.06 | - | 1.08) |  |  | **1.35** |  | (1.33 | - | 1.37) |
| **Subscales**^4^ | PF | **0.73** |  | (0.72 | - | 0.74) |  |  | **0.76** |  | (0.74 | - | 0.78) |  |  | **0.77** |  | (0.75 | - | 0.80) |  |  | **0.80** |  | (0.77 | - | 0.82) |  |  | **0.79** |  | (0.77 | - | 0.81) |  |  | **0.85** |  | (0.82 | - | 0.87) |  |  | **0.84** |  | (0.81 | - | 0.86) |  |  | **0.95** |  | (0.93 | - | 0.97) |  |  | **0.82** |  | (0.80 | - | 0.83) |
|  | RP | **0.88** |  | (0.87 | - | 0.90) |  |  | **0.77** |  | (0.75 | - | 0.79) |  |  | **0.84** |  | (0.82 | - | 0.86) |  |  | **0.85** |  | (0.83 | - | 0.86) |  |  | **0.84** |  | (0.82 | - | 0.85) |  |  | **0.92** |  | (0.90 | - | 0.95) |  |  | **0.92** |  | (0.90 | - | 0.95) |  |  | **0.97** |  | (0.96 | - | 0.99) |  |  | **0.89** |  | (0.87 | - | 0.90) |
|  | BP | **0.88** |  | (0.86 | - | 0.90) |  |  | **0.80** |  | (0.78 | - | 0.83) |  |  | **0.86** |  | (0.84 | - | 0.89) |  |  | **0.86** |  | (0.84 | - | 0.88) |  |  | **0.87** |  | (0.84 | - | 0.89) |  |  | **1.14** |  | (1.03 | - | 1.27) |  |  | **0.92** |  | (0.86 | - | 0.98) |  |  | 0.98 |  | (0.96 | - | 1.00) |  |  | **0.87** |  | (0.85 | - | 0.89) |
|  | GH | **0.87** |  | (0.84 | - | 0 90) |  |  | **0.74** |  | (0.70 | - | 0.77) |  |  | **0.79** |  | (0.75 | - | 0.83) |  |  | **0.80** |  | (0.76 | - | 0.83) |  |  | **0.81** |  | (0.77 | - | 0.84) |  |  | 0.95 |  | (0.88 | - | 1.02) |  |  | **0.92** |  | (0.86 | - | 0.98) |  |  | **1.03** |  | (1.00 | - | 1.05) |  |  | **0.86** |  | (0.84 | - | 0.88) |
|  | VT | **0.90** |  | (0.87 | - | 0.94) |  |  | **0.74** |  | (0.70 | - | 0.78) |  |  | **0.82** |  | (0.78 | - | 0.86) |  |  | **0.83** |  | (0.79 | - | 0.87) |  |  | **0.84** |  | (0.80 | - | 0.88) |  |  | 0.93 |  | (0.86 | - | 1.01) |  |  | **0.91** |  | (0.85 | - | 0.98) |  |  | 1.00 |  | (0.97 | - | 1.03) |  |  | **0.91** |  | (0.87 | - | 0.95) |
|  | SF | **0.85** |  | (0.83 | - | 0.87) |  |  | **0.76** |  | (0.74 | - | 0.78) |  |  | **0.80** |  | (0.77 | - | 0.82) |  |  | **0.81** |  | (0.79 | - | 0.83) |  |  | **0.81** |  | (0.79 | - | 0.83) |  |  | **0.65** |  | (0.63 | - | 0.69) |  |  | **0.66** |  | (0.64 | - | 0.69) |  |  | **0.96** |  | (0.94 | - | 0.98) |  |  | **0.79** |  | (0.77 | - | 0.80) |
|  | RE | **0.90** |  | (0.89 | - | 0.92) |  |  | **0.82** |  | (0.80 | - | 0.84) |  |  | **0.87** |  | (0.84 | - | 0.89) |  |  | **0.86** |  | (0.84 | - | 0.88) |  |  | **0.87** |  | (0.85 | - | 0.90) |  |  | **0.94** |  | 0.92 | - | 0.97) |  |  | **0.95** |  | (0.92 | - | 0.97) |  |  | 1.00 |  | (0.98 | - | 1.02) |  |  | **0.92** |  | (0.90 | - | 0.93) |
|  | MH | **0.91** |  | (0.88 | - | 0.95) |  |  | **0.79** |  | (0.75 | - | 0.83) |  |  | **0.85** |  | (0.81 | - | 0.90) |  |  | **0.87** |  | (0.83 | - | 0.91) |  |  | **0.86** |  | (0.82 | - | 0.91) |  |  | 0.94 |  | (0.86 | - | 1.02) |  |  | **0.90** |  | (0.84 | - | 0.98) |  |  | 0.97 |  | (0.94 | - | 1.00) |  |  | **0.88** |  | (0.85 | - | 0.92) |
|  |  |  |  |  |  |  |  |  |  |  |  |  |  |  |  |  |  |  |  |  |  |  |  |  |  |  |  |  |  |  |  |  |  |  |  |  |  |  |  |  |  |  |  |  |  |  |  |  |  |  |  |  |  |  |  |  |  |  |  |  |  |  |

OR = odds ratios; 95 % CI= 95% Confidence interval

¹ OR associated with an increment of 10 years

² Subjects without an occupation are excluded from this analysis

³ Depression as measured by the CES-D; CES-D depression score is the sum of the 20 items, further dichotomized (a score ≥ 16 indicates depression; the score is not computed if more than 4 items are missing).

^4^ OR associated with an increment of 10 points for all subscale scores

**Additional file 2:** Continued

|  | |  | | **GH3** | | | | | | | | | |  | |  | | **GH4** | | | | | | | | | |  | |  | | **GH5** | | | | | | | | | |  | |  | | **VT1** | | | | | | | | | |  | |  | | **VT2** | | | | | | | | | |  | |  | | **VT3** | | | | | | | | | | | | | | **VT4** | | | | | | | | | |  | |  | | **SF1** | | | | | | | | | |  | |  | | **SF2** | | | | | |  |
| --- | --- | --- | --- | --- | --- | --- | --- | --- | --- | --- | --- | --- | --- | --- | --- | --- | --- | --- | --- | --- | --- | --- | --- | --- | --- | --- | --- | --- | --- | --- | --- | --- | --- | --- | --- | --- | --- | --- | --- | --- | --- | --- | --- | --- | --- | --- | --- | --- | --- | --- | --- | --- | --- | --- | --- | --- | --- | --- | --- | --- | --- | --- | --- | --- | --- | --- | --- | --- | --- | --- | --- | --- | --- | --- | --- | --- | --- | --- | --- | --- | --- | --- | --- | --- | --- | --- | --- | --- | --- | --- | --- | --- | --- | --- | --- | --- | --- | --- | --- | --- | --- | --- | --- | --- | --- | --- | --- | --- | --- | --- | --- | --- | --- | --- | --- | --- | --- | --- | --- | --- | --- | --- |
|  | |  | |  | |  | |  | |  | |  | |  | |  | |  | |  | |  | |  | |  | |  | |  | |  | |  | |  | |  | |  | |  | |  | |  | |  | |  | |  | |  | |  | |  | |  | |  | |  | |  | |  | |  | |  | |  | |  | |  | |  | |  | |  | |  | |  | |  | |  | |  | |  | |  | |  | |  | |  | |  | |  | |  | |  | |  | |  | |  | |  | |  |
| **Proportion of missing data** | | | | **6.0%** | | | | | | | | | |  | |  | | **6.1%** | | | | | | | | | |  | |  | | **6.8%** | | | | | | | | | |  | |  | | **5.6%** | | | | | | | | | |  | |  | | **5.6%** | | | | | | | | | |  | |  | | **5.5%** | | | | | | | | | |  | |  | | **4.0%** | | | | | | | | | |  | |  | | **2.6%** | | | | | | | | | |  | |  | | **3.0%** | | | | | | |
|  | |  | | **OR** | |  | | **(95% CI)** | | | | | |  | |  | | **OR** | |  | | **(95% CI)** | | | | | |  | |  | | **OR** | |  | | **(95% CI)** | | | | | |  | |  | | **OR** | |  | | **(95% CI)** | | | | | |  | |  | | **OR** | |  | | **(95% CI)** | | | | | |  | |  | | **OR** | |  | | **(95% CI)** | | | | | |  | |  | | **OR** | |  | | **(95% CI)** | | | | | |  | |  | | **OR** | |  | | **(95% CI)** | | | | | |  | |  | | **OR** | |  | | **(95% CI)** | | |
|  | |  | |  | |  | |  | |  | |  | |  | |  | |  | |  | |  | |  | |  | |  | |  | |  | |  | |  | |  | |  | |  | |  | |  | |  | |  | |  | |  | |  | |  | |  | |  | |  | |  | |  | |  | |  | |  | |  | |  | |  | |  | |  | |  | |  | |  | |  | |  | |  | |  | |  | |  | |  | |  | |  | |  | |  | |  | |  | |  | |  | |  |
| **Age**¹ | |  | | **1.57** | | ( | | 1.52 | | - | | 1.62 | | ) | |  | | **1.49** | | ( | | 1.44 | | - | | 1.54 | | ) | |  | | **1.65** | | ( | | 1.60 | | - | | 1.71 | | ) | |  | | **1.65** | | ( | | 1.59 | | - | | 1.71 | | ) | |  | | **1.62** | | ( | | 1.56 | | - | | 1.68 | | ) | |  | | **1.61** | | ( | | 1.56 | | - | | 1.67 | | ) | |  | | **1.50** | | ( | | 1.45 | | - | | 1.57 | | ) | |  | | **1.42** | | ( | | 1.36 | | - | | 1.49 | | ) | |  | | **1.43** | | ( | | 1.36 | | -1.49 ) |
| **Gender** | | Male | |  | |  | |  | |  | |  | |  | |  | |  | |  | |  | |  | |  | |  | |  | |  | |  | |  | |  | |  | |  | |  | |  | |  | |  | |  | |  | |  | |  | |  | |  | |  | |  | |  | |  | |  | |  | |  | |  | |  | |  | |  | |  | |  | |  | |  | |  | |  | |  | |  | |  | |  | |  | |  | |  | |  | |  | |  | |  | |  | |  |
|  | | Female | | **1.25** | | ( | | 1.11 | | - | | 1.39 | | ) | |  | | **1.30** | | ( | | 1.16 | | - | | 1.45 | | ) | |  | | **1.35** | | ( | | 1.22 | | - | | 1.50 | | ) | |  | | **1.21** | | ( | | 1.08 | | - | | 1.35 | | ) | |  | | **1.16** | | ( | | 1.04 | | - | | 1.30 | | ) | |  | | **1.17** | | ( | | 1.04 | | - | | 1.31 | | ) | |  | | 1.09 | | ( | | 0.95 | | - | | 1.24 | | ) | |  | | **1.35** | | ( | | 1.14 | | - | | 1.60 | | ) | |  | | 1.08 | | ( | | 0.93 | | -1.26 ) |
| **Education** | | no diploma | |  | |  | |  | |  | |  | |  | |  | |  | |  | |  | |  | |  | |  | |  | |  | |  | |  | |  | |  | |  | |  | |  | |  | |  | |  | |  | |  | |  | |  | |  | |  | |  | |  | |  | |  | |  | |  | |  | |  | |  | |  | |  | |  | |  | |  | |  | |  | |  | |  | |  | |  | |  | |  | |  | |  | |  | |  | |  | |  | |  |
|  | | < HS graduate | | **0.35** | | ( | | 0.31 | | - | | 0.40 | | ) | |  | | **0.40** | | ( | | 0.35 | | - | | 0.45 | | ) | |  | | **0.34** | | ( | | 0.30 | | - | | 0.38 | | ) | |  | | **0.33** | | ( | | 0.28 | | - | | 0.37 | | ) | |  | | **0.33** | | ( | | 0.29 | | - | | 0.38 | | ) | |  | | **0.35** | | ( | | 0.30 | | - | | 0.40 | | ) | |  | | **0.41** | | ( | | 0.35 | | - | | 0.47 | | ) | |  | | **0.40** | | ( | | 0.33 | | - | | 0.49 | | ) | |  | | **0.43** | | ( | | 0.36 | | -0.51 ) |
|  | | HS graduate | | **0.28** | | ( | | 0.24 | | - | | 0.33 | | ) | |  | | **0.26** | | ( | | 0.22 | | - | | 0.31 | | ) | |  | | **0.23** | | ( | | 0.19 | | - | | 0.27 | | ) | |  | | **0.21** | | ( | | 0.18 | | - | | 0.25 | | ) | |  | | **0.22** | | ( | | 0.18 | | - | | 0.26 | | ) | |  | | **0.25** | | ( | | 0.21 | | - | | 0.29 | | ) | |  | | **0.28** | | ( | | 0.23 | | - | | 0.34 | | ) | |  | | **0.34** | | ( | | 0.27 | | - | | 0.43 | | ) | |  | | **0.32** | | ( | | 0.26 | | -0.40 ) |
|  | | university | | **0.23** | | ( | | 0.18 | | - | | 0.30 | | ) | |  | | **0.23** | | ( | | 0.18 | | - | | 0.29 | | ) | |  | | **0.17** | | ( | | 0.13 | | - | | 0.22 | | ) | |  | | **0.14** | | ( | | 0.11 | | - | | 0.19 | | ) | |  | | **0.15** | | ( | | 0.11 | | - | | 0.20 | | ) | |  | | **0.16** | | ( | | 0.12 | | - | | 0.22 | | ) | |  | | **0.20** | | ( | | 0.15 | | - | | 0.28 | | ) | |  | | **0.21** | | ( | | 0.14 | | - | | 0.31 | | ) | |  | | **0.23** | | ( | | 0.16 | | -0.32 ) |
| **Occupation**² | | white collar | |  | |  | |  | |  | |  | |  | |  | |  | |  | |  | |  | |  | |  | |  | |  | |  | |  | |  | |  | |  | |  | |  | |  | |  | |  | |  | |  | |  | |  | |  | |  | |  | |  | |  | |  | |  | |  | |  | |  | |  | |  | |  | |  | |  | |  | |  | |  | |  | |  | |  | |  | |  | |  | |  | |  | |  | |  | |  | |  | |  |
|  | | blue collar | | **1.63** | | ( | | 1.45 | | - | | 1.83 | | ) | |  | | **1.56** | | ( | | 1.39 | | - | | 1.76 | | ) | |  | | **1.60** | | ( | | 1.43 | | - | | 1.78 | | ) | |  | | **1.79** | | ( | | 1.59 | | - | | 2.02 | | ) | |  | | **1.83** | | ( | | 1.62 | | - | | 2.06 | | ) | |  | | **1.77** | | ( | | 1.56 | | - | | 1.99 | | ) | |  | | **1.58** | | ( | | 1.37 | | - | | 1.82 | | ) | |  | | **1.63** | | ( | | 1.37 | | - | | 1.94 | | ) | |  | | **1.63** | | ( | | 1.39 | | -1.91 ) |
| **French nationality** | | yes | |  | |  | |  | |  | |  | |  | |  | |  | |  | |  | |  | |  | |  | |  | |  | |  | |  | |  | |  | |  | |  | |  | |  | |  | |  | |  | |  | |  | |  | |  | |  | |  | |  | |  | |  | |  | |  | |  | |  | |  | |  | |  | |  | |  | |  | |  | |  | |  | |  | |  | |  | |  | |  | |  | |  | |  | |  | |  | |  | |  |
|  | | no | | **1.25** | | ( | | 1.04 | | - | | 1.51 | | ) | |  | | **1.31** | | ( | | 1.09 | | - | | 1.57 | | ) | |  | | 1.12 | | ( | | 0.93 | | - | | 1.35 | | ) | |  | | **1.22** | | ( | | 1.01 | | - | | 1.49 | | ) | |  | | 1.09 | | ( | | 0.89 | | - | | 1.34 | | ) | |  | | 1.01 | | ( | | 0.82 | | - | | 1.25 | | ) | |  | | 0.94 | | ( | | 0.73 | | - | | 1.21 | | ) | |  | | 1.15 | | ( | | 0.86 | | - | | 1.54 | | ) | |  | | 1.02 | | ( | | 0.77 | | -1.34 ) |
| **Chronic disease** | | no | |  | |  | |  | |  | |  | |  | |  | |  | |  | |  | |  | |  | |  | |  | |  | |  | |  | |  | |  | |  | |  | |  | |  | |  | |  | |  | |  | |  | |  | |  | |  | |  | |  | |  | |  | |  | |  | |  | |  | |  | |  | |  | |  | |  | |  | |  | |  | |  | |  | |  | |  | |  | |  | |  | |  | |  | |  | |  | |  | |  |
|  | | yes | | **2.55** | | ( | | 2.24 | | - | | 2.90 | | ) | |  | | **1.80** | | ( | | 1.5 | | - | | 2.0 | | ) | |  | | **2.72** | | ( | | 2.41 | | - | | 3.08 | | ) | |  | | **2.50** | | ( | | 2.19 | | - | | 2.86 | | ) | |  | | **2.33** | | ( | | 2.03 | | - | | 2.67 | | ) | |  | | **2.08** | | ( | | 1.81 | | - | | 2.39 | | ) | |  | | **2.01** | | ( | | 1.71 | | - | | 2.37 | | ) | |  | | **1.70** | | ( | | 1.37 | | - | | 2.10 | | ) | |  | | **1.84** | | ( | | 1.52 | | -2.22 ) |
| **Hospitalization** | | no | |  | |  | |  | |  | |  | |  | |  | |  | |  | |  | |  | |  | |  | |  | |  | |  | |  | |  | |  | |  | |  | |  | |  | |  | |  | |  | |  | |  | |  | |  | |  | |  | |  | |  | |  | |  | |  | |  | |  | |  | |  | |  | |  | |  | |  | |  | |  | |  | |  | |  | |  | |  | |  | |  | |  | |  | |  | |  | |  | |  |
| **in the year** | | yes | | **1.79** | | ( | | 1.56 | | - | | 2.05 | | ) | |  | | **1.41** | | ( | | 1.22 | | - | | 1.63 | | ) | |  | | **1.86** | | ( | | 1.63 | | - | | 2.11 | | ) | |  | | **1.54** | | ( | | 1.33 | | - | | 1.78 | | ) | |  | | **1.45** | | ( | | 1.22 | | - | | 1.68 | | ) | |  | | **1.32** | | ( | | 1.13 | | - | | 1.54 | | ) | |  | | **1.38** | | ( | | 1.16 | | - | | 1.65 | | ) | |  | | **1.25** | | ( | | 1.00 | | - | | 1.56 | | ) | |  | | **1.31** | | ( | | 1.07 | | -1.61 ) |
| **Vision disability** | | no | |  | |  | |  | |  | |  | |  | |  | |  | |  | |  | |  | |  | |  | |  | |  | |  | |  | |  | |  | |  | |  | |  | |  | |  | |  | |  | |  | |  | |  | |  | |  | |  | |  | |  | |  | |  | |  | |  | |  | |  | |  | |  | |  | |  | |  | |  | |  | |  | |  | |  | |  | |  | |  | |  | |  | |  | |  | |  | |  | |  |
|  | | yes | | 1.07 | | ( | | 0.82 | | - | | 1.39 | | ) | |  | | 1.13 | | ( | | 0.87 | | - | | 1.46 | | ) | |  | | 1.07 | | ( | | 0.84 | | - | | 1.38 | | ) | |  | | **1.39** | | ( | | 1.08 | | - | | 1.78 | | ) | |  | | 1.11 | | ( | | 0.85 | | - | | 1.45 | | ) | |  | | 1.16 | | ( | | 0.89 | | - | | 1.51 | | ) | |  | | 1.11 | | ( | | 0.81 | | - | | 1.53 | | ) | |  | | 0.94 | | ( | | 0.62 | | - | | 1.43 | | ) | |  | | 1.14 | | ( | | 0.80 | | -1.63 ) |
| **Depression**³ | | no | |  | |  | |  | |  | |  | |  | |  | |  | |  | |  | |  | |  | |  | |  | |  | |  | |  | |  | |  | |  | |  | |  | |  | |  | |  | |  | |  | |  | |  | |  | |  | |  | |  | |  | |  | |  | |  | |  | |  | |  | |  | |  | |  | |  | |  | |  | |  | |  | |  | |  | |  | |  | |  | |  | |  | |  | |  | |  | |  | |  |
|  | | yes | | **1.97** | | ( | | 1.71 | | - | | 2.27 | | ) | |  | | **1.29** | | ( | | 1.11 | | - | | 1.51 | | ) | |  | | **2.06** | | ( | | 1.80 | | - | | 2.36 | | ) | |  | | **1.67** | | ( | | 1.43 | | - | | 1.95 | | ) | |  | | **1.49** | | ( | | 1.27 | | - | | 1.75 | | ) | |  | | **1.34** | | ( | | 1.14 | | - | | 1.58 | | ) | |  | | **1.23** | | ( | | 1.00 | | - | | 1.50 | | ) | |  | | **1.65** | | ( | | 1.30 | | - | | 2.10 | | ) | |  | | 1.23 | | ( | | 0.97 | | -1.56 ) |
| **Number of missing data  for other items** | | | | **1.31** | | ( | | 1.30 | | - | | 1.32 | | ) | |  | | **1.31** | | ( | | 1.30 | | - | | 1.33 | | ) | |  | | **1.37** | | ( | | 1.36 | | - | | 1.39 | | ) | |  | | **1.42** | | ( | | 1.40 | | - | | 1.44 | | ) | |  | | **1.51** | | ( | | 1.49 | | - | | 1.53 | | ) | |  | | **1.45** | | ( | | 1.43 | | - | | 1.47 | | ) | |  | | **1.37** | | ( | | 1.35 | | - | | 1.39 | | ) | |  | | **1.24** | | ( | | 1.23 | | - | | 1.26 | | ) | |  | | **1.27** | | ( | | 1.26 | | -1.29 ) |
| **Subscales**^4^ | | PF | | **0.81** | | ( | | 0.79 | | - | | 0.82 | | ) | |  | | **0.85** | | ( | | 0.83 | | - | | 0.87 | | ) | |  | | **0.80** | | ( | | 0.79 | | - | | 0.82 | | ) | |  | | **0.82** | | ( | | 0.81 | | - | | 0.84 | | ) | |  | | **0.83** | | ( | | 0.81 | | - | | 0.85 | | ) | |  | | **0.84** | | ( | | 0.82 | | - | | 0.86 | | ) | |  | | **0.86** | | ( | | 0.84 | | - | | 0.88 | | ) | |  | | **0.83** | | ( | | 0.81 | | - | | 0.85 | | ) | |  | | **0.85** | | ( | | 0.83 | | -0.87 ) |
|  | | RP | | **0.87** | | ( | | 0.86 | | - | | 0.89 | | ) | |  | | **0.92** | | ( | | 0.90 | | - | | 0.93 | | ) | |  | | **0.87** | | ( | | 0.86 | | - | | 0.88 | | ) | |  | | **0.89** | | ( | | 0.87 | | - | | 0.90 | | ) | |  | | **0.89** | | ( | | 0.88 | | - | | 0.91 | | ) | |  | | **0.92** | | ( | | 0.90 | | - | | 0.93 | | ) | |  | | **0.93** | | ( | | 0.91 | | - | | 0.95 | | ) | |  | | **0.91** | | ( | | 0.89 | | - | | 0.94 | | ) | |  | | **0.92** | | ( | | 0.90 | | -0.94 ) |
|  | | BP | | **0.84** | | ( | | 0.82 | | - | | 0.86 | | ) | |  | | **0.94** | | ( | | 0.91 | | - | | 0.96 | | ) | |  | | **0.82** | | ( | | 0.80 | | - | | 0.84 | | ) | |  | | **0.86** | | ( | | 0.84 | | - | | 0.88 | | ) | |  | | **0.89** | | ( | | 0.86 | | - | | 0.91 | | ) | |  | | **0.93** | | ( | | 0.91 | | - | | 0.95 | | ) | |  | | **0.96** | | ( | | 0.93 | | - | | 0.99 | | ) | |  | | **0.80** | | ( | | 0.76 | | - | | 0.85 | | ) | |  | | **0.93** | | ( | | 0.89 | | -0.97 ) |
|  | | GH | | **0.72** | | ( | | 0.71 | | - | | 0.74 | | ) | |  | | **0.89** | | ( | | 0.87 | | - | | 0.91 | | ) | |  | | **0.70** | | ( | | 0.69 | | - | | 0.72 | | ) | |  | | **0.84** | | ( | | 0.80 | | - | | 0.88 | | ) | |  | | **0.89** | | ( | | 0.85 | | - | | 0.94 | | ) | |  | | **0.94** | | ( | | 0.90 | | - | | 0.99 | | ) | |  | | 0.96 | | ( | | 0.90 | | - | | 1.02 | | ) | |  | | **0.83** | | ( | | 0.78 | | - | | 0.89 | | ) | |  | | **0.91** | | ( | | 0.85 | | -0.98 ) |
|  | | VT | | **0.82** | | ( | | 0.79 | | - | | 0.86 | | ) | |  | | 0.99 | | ( | | 0.95 | | - | | 1.03 | | ) | |  | | **0.81** | | ( | | 0.78 | | - | | 0.84 | | ) | |  | | **0.78** | | ( | | 0.75 | | - | | 0.82 | | ) | |  | | **0.79** | | ( | | 0.76 | | - | | 0.82 | | ) | |  | | 0.96 | | ( | | 0.91 | | - | | 1.00 | | ) | |  | | 0.98 | | ( | | 0.91 | | - | | 1.05 | | ) | |  | | **0.83** | | ( | | 0.77 | | - | | 0.89 | | ) | |  | | **0.89** | | ( | | 0.83 | | -0.97 ) |
|  | | SF | | **0.74** | | ( | | 0.73 | | - | | 0.76 | | ) | |  | | **0.82** | | ( | | 0.80 | | - | | 0.84 | | ) | |  | | **0.75** | | ( | | 0.73 | | - | | 0.77 | | ) | |  | | **0.77** | | ( | | 0.75 | | - | | 0.78 | | ) | |  | | **0.77** | | ( | | 0.76 | | - | | 0.79 | | ) | |  | | **0.80** | | ( | | 0.78 | | - | | 0.82 | | ) | |  | | **0.77** | | ( | | 0.75 | | - | | 0.79 | | ) | |  | | **0.87** | | ( | | 0.83 | | - | | 0.92 | | ) | |  | | 1.00 | | ( | | 0.95 | | -1.05 ) |
|  | | RE | | **0.90** | | ( | | 0.89 | | - | | 0.92 | | ) | |  | | **0.95** | | ( | | 0.94 | | - | | 0.97 | | ) | |  | | **0.89** | | ( | | 0.88 | | - | | 0.90 | | ) | |  | | **0.92** | | ( | | 0.90 | | - | | 0.93 | | ) | |  | | **0.93** | | ( | | 0.91 | | - | | 0.94 | | ) | |  | | **0.95** | | ( | | 0.93 | | - | | 0.97 | | ) | |  | | **0.97** | | ( | | 0.95 | | - | | 0.99 | | ) | |  | | **0.93** | | ( | | 0.91 | | - | | 0.96 | | ) | |  | | **0.96** | | ( | | 0.94 | | -0.99 ) |
|  | | MH | | **0.83** | | ( | | 0.80 | | - | | 0.86 | | ) | |  | | **0.95** | | ( | | 0.91 | | - | | 0.99 | | ) | |  | | **0.80** | | ( | | 0.77 | | - | | 0.83 | | ) | |  | | **0.81** | | ( | | 0.76 | | - | | 0.85 | | ) | |  | | **0.89** | | ( | | 0.83 | | - | | 0.95 | | ) | |  | | 0.97 | | ( | | 0.90 | | - | | 1.04 | | ) | |  | | 0.91 | | ( | | 0.82 | | - | | 1.01 | | ) | |  | | **0.79** | | ( | | 0.74 | | - | | 0.85 | | ) | |  | | 0.93 | | ( | | 0.86 | | -1.01 ) |
|  |  | |  | |  | |  | |  | |  | |  | |  | |  | |  | |  | |  | |  | |  | |  | |  | |  | |  | |  | |  | |  | |  | |  | |  | |  | |  | |  | |  | |  | |  | |  | |  | |  | |  | |  | |  | |  | |  | |  | |  | |  | |  | |  | |  | |  | |  | |  | |  | |  | |  | |  | |  | |  | |  | |  | |  | |  | |  | |  | |  | |  | |

OR = odds ratios; 95 % CI= 95% Confidence interval

¹ OR associated with an increment of 10 years

² Subjects without an occupation are excluded from this analysis

³ Depression as measured by the CES-D; CES-D depression score is the sum of the 20 items, further dichotomized (a score ≥ 16 indicates depression; the score is not computed if more than 4 items are missing).

^4^ OR associated with an increment of 10 points for all subscale scores

**Additional file 2:** Continued

|  |  | **RE1** | | | | |  |  | **RE2** | | | | | | | **RE3** | | | | |  |  | **MH1** | | | | |  |  | **MH2** | | | | | | | **MH3** | | | | | | | **MH4** | | | | |  |  | **MH5** | | | | |  |  |
| --- | --- | --- | --- | --- | --- | --- | --- | --- | --- | --- | --- | --- | --- | --- | --- | --- | --- | --- | --- | --- | --- | --- | --- | --- | --- | --- | --- | --- | --- | --- | --- | --- | --- | --- | --- | --- | --- | --- | --- | --- | --- | --- | --- | --- | --- | --- | --- | --- | --- | --- | --- | --- | --- | --- | --- | --- | --- |
|  |  |  |  |  |  |  |  |  |  |  |  |  |  |  |  |  |  |  |  |  |  |  |  |  |  |  |  |  |  |  |  |  |  |  |  |  |  |  |  |  |  |  |  |  |  |  |  |  |  |  |  |  |  |  |  |  |  |
| **Proportion of missing data** | | **3.7%** | | | | |  |  | **3.6%** | | | | |  |  | **6.3%** | | | | |  |  | **5.0%** | | | | |  |  | **5.0%** | | | | |  |  | **5.3%** | | | | |  |  | **5.2%** | | | | |  |  | **5.2%** | | | | |  |  |
|  |  | **OR** |  | **(95% CI)** | | |  |  | **OR** |  | **(95% CI)** | | |  |  | **OR** |  | **(95% CI)** | | |  |  | **OR** |  | **(95% CI)** | | |  |  | **OR** |  | **(95% CI)** | | |  |  | **OR** |  | **(95% CI)** | | |  |  | **OR** |  | **(95% CI)** | | |  |  | **OR** |  | **(95% CI)** | | |  |  |
|  |  |  |  |  |  |  |  |  |  |  |  |  |  |  |  |  |  |  |  |  |  |  |  |  |  |  |  |  |  |  |  |  |  |  |  |  |  |  |  |  |  |  |  |  |  |  |  |  |  |  |  |  |  |  |  |  |  |
| **Age**¹ |  | **1.81** | ( | 1.73 | - | 1.89 | ) |  | **1.75** | ( | 1.67 | - | 1.83 | ) |  | **1.65** | ( | 1.58 | - | 1.72 | ) |  | **1.66** | ( | 1.60 | - | 1.72 | ) |  | **1.66** | ( | 1.60 | - | 1.72 | ) |  | **1.56** | ( | 1.51 | - | 1.62 | ) |  | **1.61** | ( | 1.56 | - | 1.67 | ) |  | **1.61** | ( | 1.55 | - | 1.66 | ) |  |
| **Gender** | Male |  |  |  |  |  |  |  |  |  |  |  |  |  |  |  |  |  |  |  |  |  |  |  |  |  |  |  |  |  |  |  |  |  |  |  |  |  |  |  |  |  |  |  |  |  |  |  |  |  |  |  |  |  |  |  |  |
|  | Female | **1.43** | ( | 1.24 | - | 1.64 | ) |  | **1.24** | ( | 1.07 | - | 1.43 | ) |  | **1.23** | ( | 1.06 | - | 1.42 | ) |  | **1.18** | ( | 1.04 | - | 1.33 | ) |  | **1.13** | ( | 1.00 | - | 1.27 | ) |  | **1.21** | ( | 1.08 | - | 1.37 | ) |  | **1.19** | ( | 1.05 | - | 1.34 | ) |  | **1.20** | ( | 1.08 | - | 1.35 | ) |  |
| **Education** | no diploma |  |  |  |  |  |  |  |  |  |  |  |  |  |  |  |  |  |  |  |  |  |  |  |  |  |  |  |  |  |  |  |  |  |  |  |  |  |  |  |  |  |  |  |  |  |  |  |  |  |  |  |  |  |  |  |  |
|  | < HS graduate | **0.25** | ( | 0.21 | - | 0.29 | ) |  | **0.23** | ( | 0.19 | - | 0.28 | ) |  | **0.26** | ( | 0.22 | - | 0.31 | ) |  | **0.34** | ( | 0.30 | - | 0.40 | ) |  | **0.33** | ( | 0.29 | - | 0.38 | ) |  | **0.35** | ( | 0.31 | - | 0.40 | ) |  | **0.34** | ( | 0.29 | - | 0.39 | ) |  | **0.35** | ( | 0.31 | - | 0.40 | ) |  |
|  | HS graduate | **0.18** | ( | 0.14 | - | 0.22 | ) |  | **0.18** | ( | 0.14 | - | 0.22 | ) |  | **0.19** | ( | 0.15 | - | 0.24 | ) |  | **0.24** | ( | 0.20 | - | 0.29 | ) |  | **0.23** | ( | 0.19 | - | 0.28 | ) |  | **0.22** | ( | 0.18 | - | 0.26 | ) |  | **0.22** | ( | 0.18 | - | 0.26 | ) |  | **0.25** | ( | 0.21 | - | 0.30 | ) |  |
|  | university | **0.09** | ( | 0.06 | - | 0.14 | ) |  | **0.10** | ( | 0.07 | - | 0.16 | ) |  | **0.11** | ( | 0.07 | - | 0.16 | ) |  | **0.15** | ( | 0.11 | - | 0.21 | ) |  | **0.17** | ( | 0.12 | - | 0.22 | ) |  | **0.17** | ( | 0.13 | - | 0.23 | ) |  | **0.17** | ( | 0.12 | - | 0.22 | ) |  | **0.16** | ( | 0.12 | - | 0.21 | ) |  |
| **Occupation**² | white collar |  |  |  |  |  |  |  |  |  |  |  |  |  |  |  |  |  |  |  |  |  |  |  |  |  |  |  |  |  |  |  |  |  |  |  |  |  |  |  |  |  |  |  |  |  |  |  |  |  |  |  |  |  |  |  |  |
|  | blue collar | **1.90** | ( | 1.65 | - | 2.20 | ) |  | **2.06** | ( | 1.79 | - | 2.39 | ) |  | **2.18** | ( | 1.87 | - | 2.54 | ) |  | **1.68** | ( | 1.48 | - | 1.91 | ) |  | **1.84** | ( | 1.62 | - | 2.09 | ) |  | **1.78** | ( | 1.57 | - | 2.02 | ) |  | **1.86** | ( | 1.64 | - | 2.11 | ) |  | **1.70** | ( | 1.51 | - | 1.91 | ) |  |
| **French nationality** | Yes |  |  |  |  |  |  |  |  |  |  |  |  |  |  |  |  |  |  |  |  |  |  |  |  |  |  |  |  |  |  |  |  |  |  |  |  |  |  |  |  |  |  |  |  |  |  |  |  |  |  |  |  |  |  |  |  |
|  | no | **1.27** | ( | 1.01 | - | 1.60 | ) |  | **1.40** | ( | 1.11 | - | 1.76 | ) |  | **1.36** | ( | 1.07 | - | 1.73 | ) |  | 0.98 | ( | 0.79 | - | 1.23 | ) |  | 1.08 | ( | 0.87 | - | 1.34 | ) |  | 1.03 | ( | 0.83 | - | 1.27 | ) |  | 1.01 | ( | 0.82 | - | 1.26 | ) |  | 1.08 | ( | 0.8 | - | 1.32 | ) |  |
| **Chronic disease** | no |  |  |  |  |  |  |  |  |  |  |  |  |  |  |  |  |  |  |  |  |  |  |  |  |  |  |  |  |  |  |  |  |  |  |  |  |  |  |  |  |  |  |  |  |  |  |  |  |  |  |  |  |  |  |  |  |
|  | yes | **3.08** | ( | 2.64 | - | 3.59 | ) |  | **3.08** | ( | 2.63 | - | 3.61 | ) |  | **2.62** | ( | 2.21 | - | 3.09 | ) |  | **2.41** | ( | 2.09 | - | 2.77 | ) |  | **2.29** | ( | 1.98 | - | 2.64 | ) |  | **2.23** | ( | 1.93 | - | 2.57 | ) |  | **2.32** | ( | 2.02 | - | 2.69 | ) |  | **2.32** | ( | 2.03 | - | 2.65 | ) |  |
| **Hospitalization** | no |  |  |  |  |  |  |  |  |  |  |  |  |  |  |  |  |  |  |  |  |  |  |  |  |  |  |  |  |  |  |  |  |  |  |  |  |  |  |  |  |  |  |  |  |  |  |  |  |  |  |  |  |  |  |  |  |
| **in the year** | yes | **1.68** | ( | 1.42 | - | 2.00 | ) |  | **1.64** | ( | 1.37 | - | 1.96 | ) |  | **1.56** | ( | 1.30 | - | 1.88 | ) |  | **1.43** | ( | 1.22 | - | 1.67 | ) |  | **1.42** | ( | 1.21 | - | 1.67 | ) |  | **1.44** | ( | 1.24 | - | 1.68 | ) |  | **1.47** | ( | 1.26 | - | 1.72 | ) |  | **1.44** | ( | 1.24 | - | 1.66 | ) |  |
| **Vision disability** | no |  |  |  |  |  |  |  |  |  |  |  |  |  |  |  |  |  |  |  |  |  |  |  |  |  |  |  |  |  |  |  |  |  |  |  |  |  |  |  |  |  |  |  |  |  |  |  |  |  |  |  |  |  |  |  |  |
|  | yes | 1.07 | ( | 0.77 | - | 1.49 | ) |  | 1.09 | ( | 0.78 | - | 1.53 | ) |  | 1.07 | ( | 0.75 | - | 1.52 | ) |  | 1.28 | ( | 0.97 | - | 1.67 | ) |  | 1.23 | ( | 0.93 | - | 1.62 | ) |  | 1.22 | ( | 0.93 | - | 1.59 | ) |  | 1.20 | ( | 0.91 | - | 1.57 | ) |  | 1.26 | ( | 0.98 | - | 1.61 | ) |  |
| **Depression**³ | no |  |  |  |  |  |  |  |  |  |  |  |  |  |  |  |  |  |  |  |  |  |  |  |  |  |  |  |  |  |  |  |  |  |  |  |  |  |  |  |  |  |  |  |  |  |  |  |  |  |  |  |  |  |  |  |  |
|  | yes | **2.56** | ( | 2.13 | - | 3.06 | ) |  | **1.84** | ( | 1.51 | - | 2.23 | ) |  | **1.56** | ( | 1.27 | - | 1.92 | ) |  | **1.38** | ( | 1.16 | - | 1.64 | ) |  | **1.35** | ( | 1.13 | - | 1.61 | ) |  | **1.51** | ( | 1.27 | - | 1.78 | ) |  | **1.32** | ( | 1.11 | - | 1.5 | ) |  | **1.40** | ( | 1.20 | - | 1.64 | ) |  |
| **Number of missing data  for other items** | | **1.29** | ( | 1.27 | - | 1.30 | ) |  | **1.30** | ( | 1.28 | - | 1.31 | ) |  | **1.30** | ( | 1.28 | - | 1.31 | ) |  | **1.44** | ( | 1.42 | - | 1.46 | ) |  | **1.50** | ( | 1.48 | - | 1.53 | ) |  | **1.41** | ( | 1.39 | - | 1.43 | ) |  | **1.47** | ( | 1.45 | - | 1.50 | ) |  | **1.44** | ( | 1.42 | - | 1.46 | ) |  |
| **Subscales**^4^ | PF | **0.75** | ( | 0.74 | - | 0.77 | ) |  | **0.77** | ( | 0.75 | - | 0.79 | ) |  | **0.78** | ( | 0.76 | - | 0.80 | ) |  | **0.83** | ( | 0.81 | - | 0.85 | ) |  | **0.82** | ( | 0.80 | - | 0.84 | ) |  | **0.83** | ( | 0.81 | - | 0.85 | ) |  | **0.82** | ( | 0.80 | - | 0.84 | ) |  | **0.84** | ( | 0.82 | - | 0.85 | ) |  |
|  | RP | **0.79** | ( | 0.77 | - | 0.81 | ) |  | **0.82** | ( | 0.80 | - | 0.84 | ) |  | **0.82** | ( | 0.79 | - | 0.84 | ) |  | **0.90** | ( | 0.88 | - | 0.91 | ) |  | **0.89** | ( | 0.88 | - | 0.91 | ) |  | **0.89** | ( | 0.88 | - | 0.91 | ) |  | **0.90** | ( | 0.88 | - | 0.91 | ) |  | **0.90** | ( | 0.89 | - | 0.92 | ) |  |
|  | BP | **0.79** | ( | 0.77 | - | 0.81 | ) |  | **0.84** | ( | 0.81 | - | 0.86 | ) |  | **0.85** | ( | 0.82 | - | 0.87 | ) |  | **0.88** | ( | 0.86 | - | 0.90 | ) |  | **0.89** | ( | 0.86 | - | 0.91 | ) |  | **0.87** | ( | 0.85 | - | 0.89 | ) |  | **0.89** | ( | 0.87 | - | 0.91 | ) |  | **0.89** | ( | 0.87 | - | 0.91 | ) |  |
|  | GH | **0.73** | ( | 0.70 | - | 0.76 | ) |  | **0.78** | ( | 0.74 | - | 0.81 | ) |  | **0.81** | ( | 0.77 | - | 0.85 | ) |  | **0.88** | ( | 0.83 | - | 0.92 | ) |  | **0.90** | ( | 0.85 | - | 0.95 | ) |  | **0.87** | ( | 0.83 | - | 0.91 | ) |  | **0.90** | ( | 0.86 | - | 0.95 | ) |  | **0.86** | ( | 0.82 | - | 0.90 | ) |  |
|  | VT | **0.74** | ( | 0.71 | - | 0.77 | ) |  | **0.78** | ( | 0.75 | - | 0.82 | ) |  | **0.81** | ( | 0.77 | - | 0.86 | ) |  | **0.82** | ( | 0.76 | - | 0.87 | ) |  | **0.84** | ( | 0.79 | - | 0.91 | ) |  | **0.84** | ( | 0.80 | - | 0.90 | ) |  | **0.75** | ( | 0.70 | - | 0.80 | ) |  | **0.81** | ( | 0.77 | - | 0.85 | ) |  |
|  | SF | **0.74** | ( | 0.73 | - | 0.77 | ) |  | **0.78** | ( | 0.76 | - | 0.80 | ) |  | **0.79** | ( | 0.77 | - | 0.81 | ) |  | **0.77** | ( | 0.75 | - | 0.79 | ) |  | **0.78** | ( | 0.76 | - | 0.80 | ) |  | **0.75** | ( | 0.73 | - | 0.77 | ) |  | **0.77** | ( | 0.75 | - | 0.79 | ) |  | **0.78** | ( | 0.76 | - | 0.80 | ) |  |
|  | RE | **0.76** | ( | 0.74 | - | 0.78 | ) |  | **0.84** | ( | 0.82 | - | 0.86 | ) |  | **0.84** | ( | 0.81 | - | 0.86 | ) |  | **0.93** | ( | 0.92 | - | 0.95 | ) |  | **0.93** | ( | 0.92 | - | 0.95 | ) |  | **0.93** | ( | 0.91 | - | 0.95 | ) |  | **0.93** | ( | 0.92 | - | 0.95 | ) |  | **0.93** | ( | 0.92 | - | 0.95 | ) |  |
|  | MH | **0.77** | ( | 0.73 | - | 0.81 | ) |  | **0.83** | ( | 0.79 | - | 0.87 | ) |  | **0.84** | ( | 0.80 | - | 0.89 | ) |  | **0.88** | ( | 0.84 | - | 0.93 | ) |  | **0.90** | ( | 0.85 | - | 0.95 | ) |  | **0.77** | ( | 0.74 | - | 0.81 | ) |  | **0.90** | ( | 0.85 | - | 0.94 | ) |  | **0.83** | ( | 0.80 | - | 0.87 | ) |  |
|  |  |  |  |  |  |  |  |  |  |  |  |  |  |  |  |  |  |  |  |  |  |  |  |  |  |  |  |  |  |  |  |  |  |  |  |  |  |  |  |  |  |  |  |  |  |  |  |  |  |  |  |  |  |  |  |  |  |

OR = odds ratios; 95 % CI= 95% Confidence interval

¹ OR associated with an increment of 10 years

² Subjects without an occupation are excluded from this analysis

³ Depression as measured by the CES-D; CES-D depression score is the sum of the 20 items, further dichotomized (a score ≥ 16 indicates depression; the score is not computed if more than 4 items are missing).

^4^ OR associated with an increment of 10 points for all subscale scores
