# Supplementary material for: Identifying type and determinants of missing items in quality of life questionnaires: Application to the SF-36 French version of the 2003 Decennial Health Survey
Source: Health Qual Life Outcomes. 2010 Feb 3;8:16. doi: 10.1186/1477-7525-8-16 (PMC2841108; doi:10.1186/1477-7525-8-16)
Supplement: Additional file 3 — Multivariate analysis for factors associated with the missingness for each item of the SF-36. [file 1477-7525-8-16-S3.DOC]

**Additional file 3:** Multiple analysis for factors associated with the missingness for each item of the SF-36 (final model).

|  |  | PF1 | | | | PF2 | | | | |  | PF3 | | | | |  | PF4 | | | | |  | PF5 | | | | |  | PF6 | | | | |  | PF7 | | | | |  | PF8 | | | | |  | PF9 | | | | |
| --- | --- | --- | --- | --- | --- | --- | --- | --- | --- | --- | --- | --- | --- | --- | --- | --- | --- | --- | --- | --- | --- | --- | --- | --- | --- | --- | --- | --- | --- | --- | --- | --- | --- | --- | --- | --- | --- | --- | --- | --- | --- | --- | --- | --- | --- | --- | --- | --- | --- | --- | --- | --- |
| Proportion of missing |  | 3.1% | | | | 3.2% | | | | |  | 3.3% | | | | |  | 3.6% | | | | |  | 4.9% | | | | |  | 3.3% | | | | |  | 3.1% | | | | |  | 4.5% | | | | |  | 2.8% | | | | |
|  |  | **OR** | **(95% CI)** | | | **OR** |  | **(95% CI)** | | |  | **OR** |  | **(95% CI)** | | |  | **OR** |  | **(95% CI)** | | |  | **OR** |  | **(95% CI)** | | |  | **OR** |  | **(95% CI)** | | |  | **OR** |  | **(95% CI)** | | |  | **OR** |  | **(95% CI)** | | |  | **OR** |  | **(95% CI)** | | |
| **Age**¹ |  | **1.43** | (1.31 | - | 1.55) | **1.15** | ( | 1.03 | - | 1.28 | ) | **1.14** | ( | 1.02 | - | 1.26 | ) | **1.32** | ( | 1.19 | - | 1.45 | ) | **1.28** | ( | 1.19 | - | 1.37 | ) | **1.10** | ( | 1.00 | - | 1.22 | ) | **1.14** | ( | 1.02 | - | 1.26 | ) | **1.46** | ( | 1.34 | - | 1.59 | ) | 0.90 | ( | 0.78 | - | 1.03) |
| **Gender** | Male |  |  |  |  |  |  |  |  |  |  |  |  |  |  |  |  |  |  |  |  |  |  |  |  |  |  |  |  |  |  |  |  |  |  |  |  |  |  |  |  |  |  |  |  |  |  |  |  |  |  |  |
|  | Female | **1.46** | (1.11 | - | 1.93) | 0.70 | ( | 0.49 | - | 1.00 | ) | 0.87 | ( | 0.62 | - | 1.23 | ) | 1.20 | ( | 0.86 | - | 1.65 | ) | 1.08 | ( | 0.87 | - | 1.35 | ) | 0.94 | ( | 0.69 | - | 1.29 | ) | 1.14 | ( | 0.80 | - | 1.62 | ) | 0.81 | ( | 0.62 | - | 1.06 | ) | 0.78 | ( | 0.50 | - | 1.20) |
| **Education** | no diploma |  |  |  |  |  |  |  |  |  |  |  |  |  |  |  |  |  |  |  |  |  |  |  |  |  |  |  |  |  |  |  |  |  |  |  |  |  |  |  |  |  |  |  |  |  |  |  |  |  |  |  |
|  | < HS graduate |  |  |  |  |  |  |  |  |  |  |  |  |  |  |  |  |  |  |  |  |  |  | 0.81 | ( | 0.62 | - | 1.05 | ) | 0.68 | ( | 0.45 | - | 1.03 | ) |  |  |  |  |  |  |  |  |  |  |  |  |  |  |  |  |  |
|  | HS graduate |  |  |  |  |  |  |  |  |  |  |  |  |  |  |  |  |  |  |  |  |  |  | **0.55** | ( | 0.38 | - | 0.78 | ) | 0.90 | ( | 0.56 | - | 1.42 | ) |  |  |  |  |  |  |  |  |  |  |  |  |  |  |  |  |  |
|  | university |  |  |  |  |  |  |  |  |  |  |  |  |  |  |  |  |  |  |  |  |  |  | 0.71 | ( | 0.46 | - | 1.09 | ) | 1.26 | ( | 0.74 | - | 2.15 | ) |  |  |  |  |  |  |  |  |  |  |  |  |  |  |  |  |  |
| **Occupation**² | white collar |  |  |  |  |  |  |  |  |  |  |  |  |  |  |  |  |  |  |  |  |  |  |  |  |  |  |  |  |  |  |  |  |  |  |  |  |  |  |  |  |  |  |  |  |  |  |  |  |  |  |  |
|  | blue collar |  |  |  |  |  |  |  |  |  |  |  |  |  |  |  |  | **1.49** | ( | 1.07 | - | 2.05 | ) | **1.05** | ( | 0.86 | - | 1.27 | ) |  |  |  |  |  |  |  |  |  |  |  |  |  |  |  |  |  |  |  |  |  |  |  |
| **French nationality** | yes |  |  |  |  |  |  |  |  |  |  |  |  |  |  |  |  |  |  |  |  |  |  |  |  |  |  |  |  |  |  |  |  |  |  |  |  |  |  |  |  |  |  |  |  |  |  |  |  |  |  |  |
|  | no |  |  |  |  |  |  |  |  |  |  |  |  |  |  |  |  |  |  |  |  |  |  |  |  |  |  |  |  | **1.73** | ( | 1.10 | - | 2.70 | ) | **2.10** | ( | 1.30 | - | 3.41 | ) |  |  |  |  |  |  |  |  |  |  |  |
| **Chronic disease** | no |  |  |  |  |  |  |  |  |  |  |  |  |  |  |  |  |  |  |  |  |  |  |  |  |  |  |  |  |  |  |  |  |  |  |  |  |  |  |  |  |  |  |  |  |  |  |  |  |  |  |  |
|  | yes |  |  |  |  |  |  |  |  |  |  |  |  |  |  |  |  |  |  |  |  |  |  |  |  |  |  |  |  |  |  |  |  |  |  |  |  |  |  |  |  |  |  |  |  |  |  | **1.68** | ( | 0.97 | - | 2.91) |
| **Hospitalization** | no |  |  |  |  |  |  |  |  |  |  |  |  |  |  |  |  |  |  |  |  |  |  |  |  |  |  |  |  |  |  |  |  |  |  |  |  |  |  |  |  |  |  |  |  |  |  |  |  |  |  |  |
| **in the year** | yes | **1.43** | (1.01 | - | 2 01) |  |  |  |  |  |  |  |  |  |  |  |  |  |  |  |  |  |  |  |  |  |  |  |  |  |  |  |  |  |  |  |  |  |  |  |  |  |  |  |  |  |  |  |  |  |  |  |
| **Vision disability** | no |  |  |  |  |  |  |  |  |  |  |  |  |  |  |  |  |  |  |  |  |  |  |  |  |  |  |  |  |  |  |  |  |  |  |  |  |  |  |  |  |  |  |  |  |  |  |  |  |  |  |  |
|  | yes |  |  |  |  |  |  |  |  |  |  |  |  |  |  |  |  |  |  |  |  |  |  |  |  |  |  |  |  |  |  |  |  |  |  |  |  |  |  |  |  |  |  |  |  |  |  |  |  |  |  |  |
| **Depression**³ | no |  |  |  |  |  |  |  |  |  |  |  |  |  |  |  |  |  |  |  |  |  |  |  |  |  |  |  |  |  |  |  |  |  |  |  |  |  |  |  |  |  |  |  |  |  |  |  |  |  |  |  |
|  | yes |  |  |  |  |  |  |  |  |  |  |  |  |  |  |  |  |  |  |  |  |  |  |  |  |  |  |  |  |  |  |  |  |  |  |  |  |  |  |  |  |  |  |  |  |  |  |  |  |  |  |  |
| **Number of missing data  for other items** | | **1.57** | (1.57 | - | 1.65) | **2.13** | ( | 2.00 | - | 2.26 | ) | **2.23** | ( | 2.10 | - | 2.38 | ) | **2.08** | ( | 1.08 | - | 2.27 | ) | **2.09** | ( | 1.98 | - | 2.20 | ) | **2.15** | ( | 2.02 | - | 2.29 | ) | **2.25** | ( | 2.10 | - | 2.40 | ) | **2.65** | ( | 2.47 | - | 2.84 | ) | **2.37** | ( | 2.20 | - | 2.56) |
| **Subscales**^4^ | PF |  |  |  |  | **0.86** | ( | 0.81 | - | 0.92 | ) | **0.92** | ( | 0.86 | - | 0.97 | ) | **0.89** | ( | 0.84 | - | 0.94 | ) | **0.86** | ( | 0.83 | - | 0.90 | ) | **0.88** | ( | 0.84 | - | 0.94 | ) | **0.86** | ( | 0.82 | - | 0.92 | ) | **0.88** | ( | 0.84 | - | 0.92 | ) | **0.83** | ( | 0.78 | - | 0.89) |
|  | RP |  |  |  |  |  |  |  |  |  |  |  |  |  |  |  |  |  |  |  |  |  |  |  |  |  |  |  |  |  |  |  |  |  |  |  |  |  |  |  |  |  |  |  |  |  |  |  |  |  |  |  |
|  | BP |  |  |  |  |  |  |  |  |  |  |  |  |  |  |  |  | 0.94 | ( | 0.87 | - | 1.01 | ) |  |  |  |  |  |  |  |  |  |  |  |  |  |  |  |  |  |  |  |  |  |  |  |  |  |  |  |  |  |
|  | GH |  |  |  |  |  |  |  |  |  |  | **0.92** | ( | 0.86 | - | 0.97 | ) |  |  |  |  |  |  |  |  |  |  |  |  |  |  |  |  |  |  |  |  |  |  |  |  |  |  |  |  |  |  |  |  |  |  |  |
|  | VT |  |  |  |  |  |  |  |  |  |  |  |  |  |  |  |  | **1.20** | ( | 1.08 | - | 1.32 | ) | **1.12** | ( | 1.06 | - | 1.19 | ) |  |  |  |  |  |  |  |  |  |  |  |  |  |  |  |  |  |  |  |  |  |  |  |
|  | SF |  |  |  |  |  |  |  |  |  |  |  |  |  |  |  |  |  |  |  |  |  |  |  |  |  |  |  |  |  |  |  |  |  |  |  |  |  |  |  |  | **1.17** | ( | 1.10 | - | 1.24 | ) |  |  |  |  |  |
|  | RE |  |  |  |  |  |  |  |  |  |  |  |  |  |  |  |  |  |  |  |  |  |  |  |  |  |  |  |  |  |  |  |  |  |  |  |  |  |  |  |  |  |  |  |  |  |  |  |  |  |  |  |
|  | MH |  |  |  |  | **0.91** |  | 0.83 | - | 1.00 |  |  |  |  |  |  |  |  |  |  |  |  |  |  |  |  |  |  |  |  |  |  |  |  |  |  |  |  |  |  |  |  |  |  |  |  |  |  |  |  |  |  |
| Harrell’s c / Hosmer-Lemeshow p value | | 0.82 / 0.10 | | | | 0.92 / 0.15 | | | | |  | 0.94 / 0.64 | | | | |  | 0.92 / 0.09 | | | | |  | 0.87 / 0.01 | | | | |  | 0.88 / 0.05 | | | | |  | 0.94 / 0.01 | | | | |  | 0.95 / 0.01 | | | | |  | 0.94 / 0.48 | | | | |
| Type of missingness |  | MAR | | | | MNAR | | | | |  | **MNAR** | | | | |  | MNAR | | | | |  | MNAR | | | | |  | MNAR | | | | |  | MNAR | | | | |  | MNAR | | | | |  | MNAR | | | | |

OR = odds ratios; 95 % CI= 95% Confidence interval

¹ OR associated with an increment of 10 years

² Subjects without an occupation are excluded from this analysis

³ Depression as measured by the CES-D; CES-D depression score is the sum of the 20 items, further dichotomized (a score ≥ 16 indicates depression; the score is not computed if more than 4 items are missing).

^4^ OR associated with an increment of 10 points for all subscale scores

**Additional file 3:** Continued

|  | |  | | PF10 | | | | | RP1 | | | | | | | | RP2 | | | | | | | | RP3 | | | | | | | | RP4 | | | | | | | | BP1 | | | | | | | | BP2 | | | | | | GH1 | | | | | | | | GH2 | | | | | | |
| --- | --- | --- | --- | --- | --- | --- | --- | --- | --- | --- | --- | --- | --- | --- | --- | --- | --- | --- | --- | --- | --- | --- | --- | --- | --- | --- | --- | --- | --- | --- | --- | --- | --- | --- | --- | --- | --- | --- | --- | --- | --- | --- | --- | --- | --- | --- | --- | --- | --- | --- | --- | --- | --- | --- | --- | --- | --- | --- | --- | --- | --- | --- | --- | --- | --- | --- | --- | --- | --- |
| Proportion of missing | |  | | 5.4% | | | | | 3.2% | | | | | | | | 3.2% | | | | | | | | 3.8% | | | | | | | | 3.5% | | | | | | | | 2.4% | | | | | | | | 2.7% | | | | | | 6.4% | | | | | | | | 6.4% | | | | | | |
|  | |  | | **OR** | **(95% CI)** | | | | **OR** |  | **(95% CI)** | | | | |  | **OR** |  | **(95% CI)** | | | | |  | **OR** |  | **(95% CI)** | | | | |  | **OR** |  | **(95% CI)** | | | |  | | **OR** |  | **(95% CI)** | | | |  | | **OR** |  | **(95% CI)** | | |  | **OR** | | |  | **(95% CI)** | | |  | **OR** | | |  | **(95% CI)** | | |
| **Age**¹ | |  | | **1.41** | (1.31 | - | 1.52) | | 1.11 | ( | 0.98 | - | 1.27 | | | ) | 1.05 | ( | 0.92 | - | 1.21 | | | ) | **1.15** | ( | 1.05 | - | 1.27 | | | ) | **1.21** | ( | 1.09 | - | 1.36 | | ) | | 0.95 | ( | 0.75 | - | 1.20 | | ) | | 1.17 | ( | 0.99 | - | 1.38 | ) | **0.91** | | | ( | 0.87 | - | 0.94 | ) | **1.28** | | | ( | 1.20 | - | 1.37) |
| **Gender** | | Male | |  |  |  |  | |  |  |  |  |  | | |  |  |  |  |  |  | | |  |  |  |  |  |  | | |  |  |  |  |  |  | |  | |  |  |  |  |  | |  | |  |  |  |  |  |  |  | | |  |  |  |  |  |  | | |  |  |  |  |
|  | | Female | | 0.84 | (0.67 | - | 1.05) | | **1.70** | ( | 1.13 | - | 2.57 | | | ) | 1.19 | ( | 0.76 | - | 1.85 | | | ) | 1.02 | ( | 0.75 | - | 1.38 | | | ) | 1.17 | ( | 0.81 | - | 1.70 | | ) | | 1.65 | ( | 0.76 | - | 3.57 | | ) | | 1.43 | ( | 0.81 | - | 2.53 | ) | 1.06 | | | ( | 0.94 | - | 1.21 | ) | 1.08 | | | ( | 0.88 | - | 1.33) |
| **Education** | | no diploma | |  |  |  |  | |  |  |  |  |  | | |  |  |  |  |  |  | | |  |  |  |  |  |  | | |  |  |  |  |  |  | |  | |  |  |  |  |  | |  | |  |  |  |  |  |  |  | | |  |  |  |  |  |  | | |  |  |  |  |
|  | | < HS graduate | |  |  |  |  | | **0.60** | ( | 0.38 | - | 0.96 | | | ) |  |  |  |  |  | | |  |  |  |  |  |  | | |  |  |  |  |  |  | |  | |  |  |  |  |  | |  | |  |  |  |  |  |  |  | | |  |  |  |  |  |  | | |  |  |  |  |
|  | | HS graduate | |  |  |  |  | | **0.25** | ( | 0.12 | - | 0.51 | | | ) |  |  |  |  |  | | |  |  |  |  |  |  | | |  |  |  |  |  |  | |  | |  |  |  |  |  | |  | |  |  |  |  |  |  |  | | |  |  |  |  |  |  | | |  |  |  |  |
|  | | university | |  |  |  |  | | **0.19** | ( | 0.06 | - | 0.63 | | | ) |  |  |  |  |  | | |  |  |  |  |  |  | | |  |  |  |  |  |  | |  | |  |  |  |  |  | |  | |  |  |  |  |  |  |  | | |  |  |  |  |  |  | | |  |  |  |  |
| **Occupation**² | | white collar | |  |  |  |  | |  |  |  |  |  | | |  |  |  |  |  |  | | |  |  |  |  |  |  | | |  |  |  |  |  |  | |  | |  |  |  |  |  | |  | |  |  |  |  |  |  |  | | |  |  |  |  |  |  | | |  |  |  |  |
|  | | blue collar | |  |  |  |  | |  |  |  |  |  | | |  |  |  |  |  |  | | |  |  |  |  |  |  | | |  |  |  |  |  |  | |  | |  |  |  |  |  | |  | |  |  |  |  |  |  |  | | |  |  |  |  |  |  | | |  |  |  |  |
| **French nationality** | | yes | |  |  |  |  | |  |  |  |  |  | | |  |  |  |  |  |  | | |  |  |  |  |  |  | | |  |  |  |  |  |  | |  | |  |  |  |  |  | |  | |  |  |  |  |  |  |  | | |  |  |  |  |  |  | | |  |  |  |  |
|  | | no | |  |  |  |  | |  |  |  |  |  | | |  |  |  |  |  |  | | |  |  |  |  |  |  | | |  | **1.93** | ( | 1.16 | - | 3.21 | | ) | |  |  |  |  |  | |  | |  |  |  |  |  |  |  | | |  |  |  |  |  |  | | |  |  |  |  |
| **Chronic disease** | | no | |  |  |  |  | |  |  |  |  |  | | |  |  |  |  |  |  | | |  |  |  |  |  |  | | |  |  |  |  |  |  | |  | |  |  |  |  |  | |  | |  |  |  |  |  |  |  | | |  |  |  |  |  |  | | |  |  |  |  |
|  | | yes | |  |  |  |  | |  |  |  |  |  | | |  |  |  |  |  |  | | |  |  |  |  |  |  | | |  |  |  |  |  |  | |  | |  |  |  |  |  | |  | |  |  |  |  |  |  |  | | |  |  |  |  |  |  | | |  |  |  |  |
| **Hospitalization** | | no | |  |  |  |  | |  |  |  |  |  | | |  |  |  |  |  |  | | |  |  |  |  |  |  | | |  |  |  |  |  |  | |  | |  |  |  |  |  | |  | |  |  |  |  |  |  |  | | |  |  |  |  |  |  | | |  |  |  |  |
| **in the year** | | yes | |  |  |  |  | |  |  |  |  |  | | |  |  |  |  |  |  | | |  |  |  |  |  |  | | |  |  |  |  |  |  | |  | |  |  |  |  |  | |  | |  |  |  |  |  |  |  | | |  |  |  |  |  |  | | |  |  |  |  |
| **Vision disability** | | no | |  |  |  |  | |  |  |  |  |  | | |  |  |  |  |  |  | | |  |  |  |  |  |  | | |  |  |  |  |  |  | |  | |  |  |  |  |  | |  | |  |  |  |  |  |  |  | | |  |  |  |  |  |  | | |  |  |  |  |
|  | | yes | |  |  |  |  | |  |  |  |  |  | | |  |  |  |  |  |  | | |  |  |  |  |  |  | | |  |  |  |  |  |  | |  | |  |  |  |  |  | |  | |  |  |  |  |  |  |  | | |  |  |  |  |  |  | | |  |  |  |  |
| **Depression**³ | | no | |  |  |  |  | |  |  |  |  |  | | |  |  |  |  |  |  | | |  |  |  |  |  |  | | |  |  |  |  |  |  | |  | |  |  |  |  |  | |  | |  |  |  |  |  |  |  | | |  |  |  |  |  |  | | |  |  |  |  |
|  | | yes | |  |  |  |  | |  |  |  |  |  | | |  |  |  |  |  |  | | |  |  |  |  |  |  | | |  |  |  |  |  |  | |  | |  |  |  |  |  | |  | |  |  |  |  |  |  | **1.30** | | | ( | 1.09 | - | 1.55 | ) |  | | |  |  |  |  |
| **Number of missing data  for other items** | | | | **2.53** | (2.37 | - | 2.70) | | **1.72** | ( | 1.56 | - | 1.88 | | | ) | **2.05** | ( | 1.85 | - | 2.26 | | | ) | **1.69** | ( | 1.56 | - | 1.83 | | | ) | **1.84** | ( | 1.68 | - | 2.01 | | ) | | **1.42** | ( | 1.16 | - | 1.75 | | ) | | **1.57** | ( | 1.36 | - | 1.82 | ) | **1.76** | | | ( | 1.69 | - | 1.84 | ) | **2.39** | | | ( | 2.26 | - | 2.53) |
| **Subscales**^4^ | | PF | | **0.84** | (0.81 | - | 0.88) | |  |  |  |  |  | | |  |  |  |  |  |  | | |  |  |  |  |  |  | | |  |  |  |  |  |  | |  | | **0.77** |  | 0.69 | - | 0.88 | |  | |  |  |  |  |  |  |  | | |  |  |  |  |  |  | | |  |  |  |  |
|  | | RP | |  |  |  |  | |  |  |  |  |  | | |  | **0.95** | ( | 0.89 | - | 0.99 | | | ) |  |  |  |  |  | | |  | **0.93** | ( | 0.89 | - | 0.97 | | ) | |  |  |  |  |  | |  | |  |  |  |  |  |  |  | | |  |  |  |  |  |  | | |  |  |  |  |
|  | | BP | |  |  |  |  | |  |  |  |  |  | | |  |  |  |  |  |  | | |  |  |  |  |  |  | | |  |  |  |  |  |  | |  | | **1.40** | ( | 1.17 | - | 1.70 | | ) | |  |  |  |  |  |  |  | | |  |  |  |  |  |  | | |  |  |  |  |
|  | | GH | |  |  |  |  | |  |  |  |  |  | | |  | **0.84** | ( | 0.75 | - | 0.94 | | | ) | **0.90** | ( | 0.82 | - | 0.97 | | | ) |  |  |  |  |  | |  | |  |  |  |  |  | |  | |  |  |  |  |  |  |  | | |  |  |  |  |  | **1.13** | | | ( | 1.07 | - | 1.20) |
|  | | VT | | **1.20** | (1.13 | - | 1.29) | |  |  |  |  |  | | |  |  |  |  |  |  | | |  |  |  |  |  |  | | |  |  |  |  |  |  | |  | |  |  |  |  |  | |  | |  |  |  |  |  |  |  | | |  |  |  |  |  |  | | |  |  |  |  |
|  | | SF | |  |  |  |  | |  |  |  |  |  | | |  |  |  |  |  |  | | |  |  |  |  |  |  | | |  |  |  |  |  |  | |  | |  |  |  |  |  | |  | |  |  |  |  |  |  | **1.07** | | | ( | 1.03 | - | 1.10 | ) | **0.92** | | | ( | 0.87 | - | 0.96) |
|  | | RE | |  |  |  |  | | **0.89** | ( | 0.85 | - | 0.93 | | | ) |  |  |  |  |  | | |  | **0.93** | ( | 0.90 | - | 0.97 | | | ) |  |  |  |  |  | |  | |  |  |  |  |  | |  | |  |  |  |  |  |  |  | | |  |  |  |  |  |  | | |  |  |  |  |
|  | | MH | |  |  |  |  | |  |  |  |  |  | | |  |  |  |  |  |  | | |  |  |  |  |  |  | | |  |  |  |  |  |  | |  | |  |  |  |  |  | |  | |  |  |  |  |  |  |  | | |  |  |  |  |  |  | | |  |  |  |  |
| Harrell’s c / Hosmer-Lemeshow p value | | | 0.92 / 0.01 | | | | | 0.87 / 0.01 | | | | | |  | 0.89 / 0.02 | | | | | | |  | 0.77 / < 0.01 | | | | | | |  | 0.82 / 0.01 | | | | | | |  | | 0.72 / 0.82 | | | | | |  | | 0.64 / 0.78 | | | | | | | |  | 0.87 / < 0.01 | | | | | | |  | 0.94 / < 0.01 | | | | |
| Type of missingness |  | | MNAR | | | | | MAR | | | | | |  | **MNAR** | | | | | | |  | MAR | | | | | | |  | MNAR | | | | | | |  | | MNAR | | | | | |  | | MAR | | | | | | | |  | MAR | | | | | | |  | MNAR | | | | |

OR = odds ratios; 95 % CI= 95% Confidence interval

¹ OR associated with an increment of 10 years

² Subjects without an occupation are excluded from this analysis

³ Depression as measured by the CES-D; CES-D depression score is the sum of the 20 items, further dichotomized (a score ≥ 16 indicates depression; the score is not computed if more than 4 items are missing).

^4^ OR associated with an increment of 10 points for all subscale scores

**Additional file 3:** Continued

|  |  | **GH3** | | | | | | **GH4** | | | | | | **GH5** | | | | | | **VT1** | | | | | | **VT2** | | | | | | **VT3** | | | | | | **VT4** | | | | | | **SF1** | | | | | | **SF2** | | | | |
| --- | --- | --- | --- | --- | --- | --- | --- | --- | --- | --- | --- | --- | --- | --- | --- | --- | --- | --- | --- | --- | --- | --- | --- | --- | --- | --- | --- | --- | --- | --- | --- | --- | --- | --- | --- | --- | --- | --- | --- | --- | --- | --- | --- | --- | --- | --- | --- | --- | --- | --- | --- | --- | --- | --- |
| Proportion of missing |  | 6.0% | | | | | | 6.1% | | | | | | 6.8% | | | | | | 5.6% | | | | | | 5.6% | | | | | | 5.5% | | | | | | 4.0% | | | | | | 2.6% | | | | | | 3.0% | | | | |
|  |  | **OR** |  | **(95% CI)** | | |  | **OR** |  | **(95% CI)** | | |  | **OR** |  | **(95% CI)** | | |  | **OR** |  | **(95% CI)** | | |  | **OR** |  | **(95% CI)** | | |  | **OR** |  | **(95% CI)** | | |  | **OR** |  | **(95% CI)** | | |  | **OR** |  | **(95% CI)** | | |  | **OR** |  | **(95% CI)** | | |
| **Age**¹ |  | **1.07** | ( | 1.00 | - | 1.14 | ) | **1.05** | ( | 0.98 | - | 1.12 | ) | **1.16** | ( | 1.10 | - | 1.24 | ) | **1.23** | ( | 1.13 | - | 1.35 | ) | **1.09** | ( | 0.98 | - | 1.21 | ) | **1.29** | ( | 1.17 | - | 1.42 | ) | 0.89 | ( | 0.76 | - | 1.05 | ) | 0.90 | ( | 0.75 | - | 1.05 | ) | **1.19** | ( | 1.03 | - | 1.38) |
| **Gender** | Male |  |  |  |  |  |  |  |  |  |  |  |  |  |  |  |  |  |  |  |  |  |  |  |  |  |  |  |  |  |  |  |  |  |  |  |  |  |  |  |  |  |  |  |  |  |  |  |  |  |  |  |  |  |
|  | Female | 1.17 | ( | 0.96 | - | 1.44 | ) | **1.35** | ( | 1.10 | - | 1.66 | ) | **1.32** | ( | 1.07 | - | 1.61 | ) | 1.07 | ( | 0.81 | - | 1.41 | ) | 1.08 | ( | 0.77 | - | 1.57 | ) | 1.14 | ( | 0.83 | - | 1.56 | ) | 1.12 | ( | 0.67 | - | 1.86 | ) | **2.14** | ( | 1.13 | - | 4.06 | ) | 0.97 | ( | 0.59 | - | 1.58) |
| **Education** | no diploma |  |  |  |  |  |  |  |  |  |  |  |  |  |  |  |  |  |  |  |  |  |  |  |  |  |  |  |  |  |  |  |  |  |  |  |  |  |  |  |  |  |  |  |  |  |  |  |  |  |  |  |  |  |
|  | < HS graduate |  |  |  |  |  |  |  |  |  |  |  |  |  |  |  |  |  |  | 0.76 | ( | 0.55 | - | 1.07 | ) |  |  |  |  |  |  |  |  |  |  |  |  |  |  |  |  |  |  |  |  |  |  |  |  |  |  |  |  |  |
|  | HS graduate |  |  |  |  |  |  |  |  |  |  |  |  |  |  |  |  |  |  | **0.46** | ( | 0.29 | - | 0.72 | ) |  |  |  |  |  |  |  |  |  |  |  |  |  |  |  |  |  |  |  |  |  |  |  |  |  |  |  |  |  |
|  | university |  |  |  |  |  |  |  |  |  |  |  |  |  |  |  |  |  |  | **0.55** | ( | 0.31 | - | 0.97 | ) |  |  |  |  |  |  |  |  |  |  |  |  |  |  |  |  |  |  |  |  |  |  |  |  |  |  |  |  |  |
| **Occupation**² | white collar |  |  |  |  |  |  |  |  |  |  |  |  |  |  |  |  |  |  |  |  |  |  |  |  |  |  |  |  |  |  |  |  |  |  |  |  |  |  |  |  |  |  |  |  |  |  |  |  |  |  |  |  |  |
|  | blue collar |  |  |  |  |  |  |  |  |  |  |  |  |  |  |  |  |  |  |  |  |  |  |  |  | **1.55** | ( | 1.08 | - | 2.24 | ) |  |  |  |  |  |  |  |  |  |  |  |  |  |  |  |  |  |  |  |  |  |  |  |
| **French nationality** | yes |  |  |  |  |  |  |  |  |  |  |  |  |  |  |  |  |  |  |  |  |  |  |  |  |  |  |  |  |  |  |  |  |  |  |  |  |  |  |  |  |  |  |  |  |  |  |  |  |  |  |  |  |  |
|  | no |  |  |  |  |  |  | **1.39** | ( | 1.01 | - | 1.91 | ) |  |  |  |  |  |  |  |  |  |  |  |  |  |  |  |  |  |  |  |  |  |  |  |  |  |  |  |  |  |  |  |  |  |  |  |  |  |  |  |  |  |
| **Chronic disease** | no |  |  |  |  |  |  |  |  |  |  |  |  |  |  |  |  |  |  |  |  |  |  |  |  |  |  |  |  |  |  |  |  |  |  |  |  |  |  |  |  |  |  |  |  |  |  |  |  |  |  |  |  |  |
|  | yes |  |  |  |  |  |  |  |  |  |  |  |  |  |  |  |  |  |  |  |  |  |  |  |  |  |  |  |  |  |  |  |  |  |  |  |  | 1.83 | ( | 0.94 | - | 3.55 | ) |  |  |  |  |  |  |  |  |  |  |  |
| **Hospitalization** | no |  |  |  |  |  |  |  |  |  |  |  |  |  |  |  |  |  |  |  |  |  |  |  |  |  |  |  |  |  |  |  |  |  |  |  |  |  |  |  |  |  |  |  |  |  |  |  |  |  |  |  |  |  |
| **in the year** | yes | **1.50** | ( | 1.17 | - | 1.92 | ) |  |  |  |  |  |  | **1.48** | ( | 1.15 | - | 1.89 | ) |  |  |  |  |  |  |  |  |  |  |  |  |  |  |  |  |  |  |  |  |  |  |  |  |  |  |  |  |  |  |  |  |  |  |  |
| **Vision disability** | no |  |  |  |  |  |  |  |  |  |  |  |  |  |  |  |  |  |  |  |  |  |  |  |  |  |  |  |  |  |  |  |  |  |  |  |  |  |  |  |  |  |  |  |  |  |  |  |  |  |  |  |  |  |
|  | yes |  |  |  |  |  |  |  |  |  |  |  |  |  |  |  |  |  |  | **2.46** | ( | 1.54 | - | 3.92 | ) |  |  |  |  |  |  |  |  |  |  |  |  |  |  |  |  |  |  |  |  |  |  |  |  |  |  |  |  |  |
| **Depression**³ | no |  |  |  |  |  |  |  |  |  |  |  |  |  |  |  |  |  |  |  |  |  |  |  |  |  |  |  |  |  |  |  |  |  |  |  |  |  |  |  |  |  |  |  |  |  |  |  |  |  |  |  |  |  |
|  | yes |  |  |  |  |  |  |  |  |  |  |  |  |  |  |  |  |  |  | **1.60** | ( | 1.17 | - | 2.18 | ) |  |  |  |  |  |  | 1.46 | ( | 0.99 | - | 2.16 | ) |  |  |  |  |  |  |  |  |  |  |  |  |  |  |  |  |  |
| **Number of missing data  for other items** | | **2.15** | ( | 2.04 | - | 2.28 | ) | **2.46** | ( | 2.32 | - | 2.60 | ) | **2.47** | ( | 2.35 | - | 2.63 | ) | **1.56** | ( | 1.44 | - | 1.68 | ) | **1.83** | ( | 1.68 | - | 1.99 | ) | **1.75** | ( | 1.61 | - | 1.89 | ) | **2.07** | ( | 1.86 | - | 2.31 | ) | **1.57** | ( | 1.33 | - | 1.85 | ) | **1.57** | ( | 1.37 | - | 1.80) |
| **Subscales**^4^ | PF |  |  |  |  |  |  |  |  |  |  |  |  |  |  |  |  |  |  |  |  |  |  |  |  |  |  |  |  |  |  |  |  |  |  |  |  |  |  |  |  |  |  |  |  |  |  |  |  |  |  |  |  |  |
|  | RP |  |  |  |  |  |  |  |  |  |  |  |  |  |  |  |  |  |  |  |  |  |  |  |  |  |  |  |  |  |  |  |  |  |  |  |  |  |  |  |  |  |  |  |  |  |  |  |  |  |  |  |  |  |
|  | BP |  |  |  |  |  |  |  |  |  |  |  |  |  |  |  |  |  |  |  |  |  |  |  |  |  |  |  |  |  |  | **1.14** | ( | 1.06 | - | 1.22 | ) |  |  |  |  |  |  |  |  |  |  |  |  |  |  |  |  |  |
|  | GH | **0.82** | ( | 0.79 | - | 0.87 | ) |  |  |  |  |  |  | **0.85** | ( | 0.80 | - | 0.90 | ) |  |  |  |  |  |  |  |  |  |  |  |  |  |  |  |  |  |  |  |  |  |  |  |  | **0.80** | ( | 0.70 | - | 0.92 | ) |  |  |  |  |  |
|  | VT |  |  |  |  |  |  |  |  |  |  |  |  |  |  |  |  |  |  |  |  |  |  |  |  |  |  |  |  |  |  |  |  |  |  |  |  |  |  |  |  |  |  |  |  |  |  |  |  |  |  |  |  |  |
|  | SF |  |  |  |  |  |  |  |  |  |  |  |  | **0.95** | ( | 0.91 | - | 0.99 | ) |  |  |  |  |  |  |  |  |  |  |  |  |  |  |  |  |  |  |  |  |  |  |  |  |  |  |  |  |  |  |  |  |  |  |  |
|  | RE |  |  |  |  |  |  |  |  |  |  |  |  |  |  |  |  |  |  |  |  |  |  |  |  |  |  |  |  |  |  |  |  |  |  |  |  |  |  |  |  |  |  |  |  |  |  |  |  |  |  |  |  |  |
|  | MH |  |  |  |  |  |  |  |  |  |  |  |  |  |  |  |  |  |  |  |  |  |  |  |  |  |  |  |  |  |  |  |  |  |  |  |  |  |  |  |  |  |  |  |  |  |  |  |  |  |  |  |  |  |
| Harrell’s c / Hosmer-Lemeshow p value | | 0.88 / < 0.01 | | | | | | 0.92 / < 0.01 | | | | | | 0.94 / 0.01 | | | | | | 0.77 / 0.53 | | | | | | 0.75 / 0.33 | | | | | | 0.75 / 0.71 | | | | | | 0.83 / 0.04 | | | | | | 0.70 / 0.60 | | | | | | 0.64 / 0.11 | | | | |
| Type of missingness |  | **MNAR** | | | | | | **MAR** | | | | | | **MNAR** | | | | | | **MAR** | | | | | | **MAR** | | | | | | **MAR** | | | | | | **MAR** | | | | | | **MAR** | | | | | | **MAR** | | | | |

OR = odds ratios; 95 % CI= 95% Confidence interval

¹ OR associated with an increment of 10 years

² Subjects without an occupation are excluded from this analysis

³ Depression as measured by the CES-D; CES-D depression score is the sum of the 20 items, further dichotomized (a score ≥ 16 indicates depression; the score is not computed if more than 4 items are missing).

^4^ OR associated with an increment of 10 points for all subscale scores

**Additional file 3:** Continued

|  |  | **RE1** | | | | | | | **RE2** | | | | | | | **RE3** | | | | | | | **MH1** | | | | | | | **MH2** | | | | | | | **MH3** | | | | | | | **MH4** | | | | | | | **MH5** | | | | | |
| --- | --- | --- | --- | --- | --- | --- | --- | --- | --- | --- | --- | --- | --- | --- | --- | --- | --- | --- | --- | --- | --- | --- | --- | --- | --- | --- | --- | --- | --- | --- | --- | --- | --- | --- | --- | --- | --- | --- | --- | --- | --- | --- | --- | --- | --- | --- | --- | --- | --- | --- | --- | --- | --- | --- | --- | --- |
| Proportion of missing |  | **3.7%** | | | | | | | **3.6%** | | | | | | | **6.3%** | | | | | | | **5.0%** | | | | | | | **5.0%** | | | | | | | **5.3%** | | | | | | | **5.2%** | | | | | | | **5.2%** | | | | | |
|  |  | **OR** |  | **(95% CI)** | | |  |  | **OR** |  | **(95% CI)** | | |  |  | **OR** |  | **(95% CI)** | | |  |  | **OR** |  | **(95% CI)** | | |  |  | **OR** |  | **(95% CI)** | | |  |  | **OR** |  | **(95% CI)** | | |  |  | **OR** |  | **(95% CI)** | | |  |  | **OR** |  | **(95% CI)** | | |  |
| **Age**¹ |  | **1.26** | ( | 1.08 | - | 1.48 | ) |  | **1.21** | ( | 1.04 | - | 1.40 | ) |  | 0.99 | ( | 0.83 | - | 1.17 | ) |  | **1.34** | ( | 1.20 | - | 1.50 | ) |  | **1.22** | ( | 1.06 | - | 1.40 | ) |  | 1.03 | ( | 0.93 | - | 1.14 | ) |  | 1.06 | ( | 0.94 | - | 1.19 | ) |  | **1.18** | ( | 1.08 | - | 1.28 | ) |
| **Gender** | Male |  |  |  |  |  |  |  |  |  |  |  |  |  |  |  |  |  |  |  |  |  |  |  |  |  |  |  |  |  |  |  |  |  |  |  |  |  |  |  |  |  |  |  |  |  |  |  |  |  |  |  |  |  |  |  |
|  | Female | 1.36 | ( | 0.79 | - | 2.34 | ) |  | 0.74 | ( | 0.45 | - | 1.22 | ) |  | 0.94 | ( | 0.53 | - | 1.64 | ) |  | 1.07 | ( | 0.74 | - | 1.56 | ) |  | 0.90 | ( | 0.57 | - | 1.41 | ) |  | 1.10 | ( | 0.81 | - | 1.49 | ) |  | **1.50** | ( | 1.00 | - | 2.25 | ) |  | **1.31** | ( | 1.00 | - | 1.71 | ) |
| **Education** | no diploma |  |  |  |  |  |  |  |  |  |  |  |  |  |  |  |  |  |  |  |  |  |  |  |  |  |  |  |  |  |  |  |  |  |  |  |  |  |  |  |  |  |  |  |  |  |  |  |  |  |  |  |  |  |  |  |
|  | < HS graduate |  |  |  |  |  |  |  |  |  |  |  |  |  |  |  |  |  |  |  |  |  |  |  |  |  |  |  |  |  |  |  |  |  |  |  | 0.70 | ( | 0.48 | - | 1.02 | ) |  |  |  |  |  |  |  |  |  |  |  |  |  |  |
|  | HS graduate |  |  |  |  |  |  |  |  |  |  |  |  |  |  |  |  |  |  |  |  |  |  |  |  |  |  |  |  |  |  |  |  |  |  |  | **0.46** | ( | 0.29 | - | 0.75 | ) |  |  |  |  |  |  |  |  |  |  |  |  |  |  |
|  | university |  |  |  |  |  |  |  |  |  |  |  |  |  |  |  |  |  |  |  |  |  |  |  |  |  |  |  |  |  |  |  |  |  |  |  | **0.54** | ( | 0.30 | - | 0.97 | ) |  |  |  |  |  |  |  |  |  |  |  |  |  |  |
| **Occupation**² | white collar |  |  |  |  |  |  |  |  |  |  |  |  |  |  |  |  |  |  |  |  |  |  |  |  |  |  |  |  |  |  |  |  |  |  |  |  |  |  |  |  |  |  |  |  |  |  |  |  |  |  |  |  |  |  |  |
|  | blue collar |  |  |  |  |  |  |  |  |  |  |  |  |  |  | **1.83** | ( | 1.03 | - | 3.25 | ) |  |  |  |  |  |  |  |  |  |  |  |  |  |  |  |  |  |  |  |  |  |  |  |  |  |  |  |  |  |  |  |  |  |  |  |
| **French nationality** | yes |  |  |  |  |  |  |  |  |  |  |  |  |  |  |  |  |  |  |  |  |  |  |  |  |  |  |  |  |  |  |  |  |  |  |  |  |  |  |  |  |  |  |  |  |  |  |  |  |  |  |  |  |  |  |  |
|  | no |  |  |  |  |  |  |  | 1.92 | ( | 0.98 | - | 3.76 | ) |  |  |  |  |  |  |  |  |  |  |  |  |  |  |  |  |  |  |  |  |  |  |  |  |  |  |  |  |  |  |  |  |  |  |  |  |  |  |  |  |  |  |
| **Chronic disease** | no |  |  |  |  |  |  |  |  |  |  |  |  |  |  |  |  |  |  |  |  |  |  |  |  |  |  |  |  |  |  |  |  |  |  |  |  |  |  |  |  |  |  |  |  |  |  |  |  |  |  |  |  |  |  |  |
|  | yes |  |  |  |  |  |  |  |  |  |  |  |  |  |  |  |  |  |  |  |  |  |  |  |  |  |  |  |  |  |  |  |  |  |  |  |  |  |  |  |  |  |  |  |  |  |  |  |  |  |  |  |  |  |  |  |
| **Hospitalization** | no |  |  |  |  |  |  |  |  |  |  |  |  |  |  |  |  |  |  |  |  |  |  |  |  |  |  |  |  |  |  |  |  |  |  |  |  |  |  |  |  |  |  |  |  |  |  |  |  |  |  |  |  |  |  |  |
| **in the year** | yes |  |  |  |  |  |  |  |  |  |  |  |  |  |  |  |  |  |  |  |  |  |  |  |  |  |  |  |  |  |  |  |  |  |  |  |  |  |  |  |  |  |  |  |  |  |  |  |  |  |  |  |  |  |  |  |
| **Vision disability** | no |  |  |  |  |  |  |  |  |  |  |  |  |  |  |  |  |  |  |  |  |  |  |  |  |  |  |  |  |  |  |  |  |  |  |  |  |  |  |  |  |  |  |  |  |  |  |  |  |  |  |  |  |  |  |  |
|  | yes |  |  |  |  |  |  |  |  |  |  |  |  |  |  |  |  |  |  |  |  |  |  |  |  |  |  |  |  |  |  |  |  |  |  |  | **1.53** | ( | 1.00 | - | 2.79 | ) |  |  |  |  |  |  |  |  |  |  |  |  |  |  |
| **Depression**³ | no |  |  |  |  |  |  |  |  |  |  |  |  |  |  |  |  |  |  |  |  |  |  |  |  |  |  |  |  |  |  |  |  |  |  |  |  |  |  |  |  |  |  |  |  |  |  |  |  |  |  |  |  |  |  |  |
|  | yes |  |  |  |  |  |  |  |  |  |  |  |  |  |  |  |  |  |  |  |  |  |  |  |  |  |  |  |  |  |  |  |  |  |  |  |  |  |  |  |  |  |  | **0.47** | ( | 0.28 | - | 0.81 | ) |  |  |  |  |  |  |  |
| **Number of missing data  for other items** | | **1.74** | ( | 1.54 | - | 1.95 | ) |  | **1.90** | ( | 1.71 | - | 2.10 | ) |  | **1.86** | ( | 1.66 | - | 2.10 | ) |  | **1.63** | ( | 1.48 | - | 1.78 | ) |  | **1.80** | ( | 1.62 | - | 2.00 | ) |  | **1.61** | ( | 1.48 | - | 1.75 | ) |  | **1.72** | ( | 1.56 | - | 1.90 | ) |  | **1.66** | ( | 1.54 | - | 1.78 | ) |
| **Subscales**^4^ | PF |  |  |  |  |  |  |  |  |  |  |  |  |  |  |  |  |  |  |  |  |  |  |  |  |  |  |  |  |  |  |  |  |  |  |  |  |  |  |  |  |  |  |  |  |  |  |  |  |  |  |  |  |  |  |  |
|  | RP |  |  |  |  |  |  |  |  |  |  |  |  |  |  |  |  |  |  |  |  |  |  |  |  |  |  |  |  |  |  |  |  |  |  |  |  |  |  |  |  |  |  |  |  |  |  |  |  |  |  |  |  |  |  |  |
|  | BP |  |  |  |  |  |  |  |  |  |  |  |  |  |  |  |  |  |  |  |  |  |  |  |  |  |  |  |  |  |  |  |  |  |  |  |  |  |  |  |  |  |  |  |  |  |  |  |  |  |  |  |  |  |  |  |
|  | GH | **0.84** | ( | 0.73 | - | 0.96 | ) |  |  |  |  |  |  |  |  |  |  |  |  |  |  |  |  |  |  |  |  |  |  |  |  |  |  |  |  |  |  |  |  |  |  |  |  |  |  |  |  |  |  |  | **0.91** | ( | 0.85 | - | 0.98 | ) |
|  | VT |  |  |  |  |  |  |  | **0.81** | ( | 0.71 | - | 0.92 | ) |  |  |  |  |  |  |  |  |  |  |  |  |  |  |  |  |  |  |  |  |  |  |  |  |  |  |  |  |  | **0.74** | ( | 0.66 | - | 0.83 | ) |  |  |  |  |  |  |  |
|  | SF |  |  |  |  |  |  |  |  |  |  |  |  |  |  |  |  |  |  |  |  |  | **0.93** | ( | 0.86 | - | 0.99 | ) |  |  |  |  |  |  |  |  |  |  |  |  |  |  |  |  |  |  |  |  |  |  |  |  |  |  |  |  |
|  | RE | **0.86** | ( | 0.80 | - | 0.92 | ) |  |  |  |  |  |  |  |  | **0.88** | ( | 0.83 | - | 0.94 | ) |  |  |  |  |  |  |  |  |  |  |  |  |  |  |  |  |  |  |  |  |  |  |  |  |  |  |  |  |  |  |  |  |  |  |  |
|  | MH |  |  |  |  |  |  |  |  |  |  |  |  |  |  |  |  |  |  |  |  |  |  |  |  |  |  |  |  |  |  |  |  |  |  |  |  |  |  |  |  |  |  |  |  |  |  |  |  |  |  |  |  |  |  |  |
| Harrell’s c / Hosmer-Lemeshow p value | | 0.87 / 0.33 | | | | | | | 0.85 / 0.44 | | | | | | | 0.79 / 0.40 | | | | | | | 0.75 / 0.31 | | | | | | | 0.79 / 0.44 | | | | | | | 0.70 / 0.88 | | | | | | | 0.77 / 0.83 | | | | | | | 0.74 / 0.24 | | | | | |
| Type of missingness |  | **MNAR** | | | | | | | **MAR** | | | | | | | **MNAR** | | | | | | | **MAR** | | | | | | | **MAR** | | | | | | | **MAR** | | | | | | | **MAR** | | | | | | | **MAR** | | | | | |

OR = odds ratios; 95 % CI= 95% Confidence interval

¹ OR associated with an increment of 10 years

² Subjects without an occupation are excluded from this analysis

³ Depression as measured by the CES-D; CES-D depression score is the sum of the 20 items, further dichotomized (a score ≥ 16 indicates depression; the score is not computed if more than 4 items are missing).

^4^ OR associated with an increment of 10 points for all subscale scores
